# Galectin-3 promotes Aβ oligomerization and Aβ toxicity in a mouse model of Alzheimer’s disease

**DOI:** 10.1038/s41418-019-0348-z

**Published:** 2019-05-24

**Authors:** Chih-Chieh Tao, Kuang-Min Cheng, Yun-Li Ma, Wei-Lun Hsu, Yan-Chu Chen, Jong-Ling Fuh, Wei-Ju Lee, Chih-Chang Chao, Eminy H. Y. Lee

**Affiliations:** 1 Institute of Biomedical Sciences, Academia Sinica, Taipei, Taiwan; 2 Graduate Institute of Life Sciences, National Defense Medical Center, Taipei, Taiwan; 3 Institute of Neuroscience, National Cheng-chi University, Taipei, Taiwan; 4 Faculty of Medicine, National Yang-Ming University School of Medicine, Taipei, Taiwan; 5 Department of Neurology, Neurological Institute, Taipei Veterans General Hospital, Taipei, Taiwan; 6 Neurological Institute, Taichung Veterans General Hospital, Taichung, Taiwan; 7 Institute of Clinical Medicine, National Yang-Ming University School of Medicine, Taipei, Taiwan

**Keywords:** Neural ageing, Ageing

## Abstract

Amyloid-β (Aβ) oligomers largely initiate the cascade underlying the pathology of Alzheimer’s disease (AD). Galectin-3 (Gal-3), which is a member of the galectin protein family, promotes inflammatory responses and enhances the homotypic aggregation of cancer cells. Here, we examined the role and action mechanism of Gal-3 in Aβ oligomerization and Aβ toxicities. Wild-type (WT) and Gal-3-knockout (KO) mice, APP/PS1;WT mice, APP/PS1;Gal-3^+/−^ mice and brain tissues from normal subjects and AD patients were used. We found that Aβ oligomerization is reduced in Gal-3 KO mice injected with Aβ, whereas overexpression of Gal-3 enhances Aβ oligomerization in the hippocampi of Aβ-injected mice. Gal-3 expression shows an age-dependent increase that parallels endogenous Aβ oligomerization in APP/PS1 mice. Moreover, Aβ oligomerization, Iba1 expression, GFAP expression and amyloid plaque accumulation are reduced in APP/PS1;Gal-3^+/−^ mice compared with APP/PS1;WT mice. APP/PS1;Gal-3^+/−^ mice also show better acquisition and retention performance compared to APP/PS1;WT mice. In studying the mechanism underlying Gal-3-promoted Aβ oligomerization, we found that Gal-3 primarily co-localizes with Iba1, and that microglia-secreted Gal-3 directly interacts with Aβ. Gal-3 also interacts with triggering receptor expressed on myeloid cells-2, which then mediates the ability of Gal-3 to activate microglia for further Gal-3 expression. Immunohistochemical analyses show that the distribution of Gal-3 overlaps with that of endogenous Aβ in APP/PS1 mice and partially overlaps with that of amyloid plaque. Moreover, the expression of the Aβ-degrading enzyme, neprilysin, is increased in Gal-3 KO mice and this is associated with enhanced integrin-mediated signaling. Consistently, Gal-3 expression is also increased in the frontal lobe of AD patients, in parallel with Aβ oligomerization. Because Gal-3 expression is dramatically increased as early as 3 months of age in APP/PS1 mice and anti-Aβ oligomerization is believed to protect against Aβ toxicity, Gal-3 could be considered a novel therapeutic target in efforts to combat AD.

## Introduction

Senile plaque is one of the two pathological hallmarks of Alzheimer’s disease (AD), and amyloid-β peptides are the major components of senile plaques. Aβ is generated from sequential and proteolytic cleavages of the amyloid precursor protein (APP) by β-secretase and γ-secretase [1]. Individual Aβ42 monomers form soluble oligomers of different molecular weight [2], and these oligomers further aggregate to form insoluble Aβ fibrils and amyloid plaques [3]. The evidence indicates that Aβ oligomers perturb the integrity of membrane lipid bilayers, increase ion permeability, cause calcium influx and consequently affect synaptic transmission and neuronal viability [4, 5]. Conversely, disaggregation of Aβ oligomers decreases Aβ-induced inflammation and rescues cognitive deficits in APP/PS1 mice [6]. Thus, soluble Aβ oligomers are believed to represent key structures that produce cytotoxicity, contribute to synaptic deficits and initiate the detrimental cascade involved in the pathology of AD [5, 7, 8]. Soluble Aβ oligomers are therefore considered to be viable drug targets for the treatment of AD [5, 9].

Galectin-3 (Gal-3) is a member of the galectin protein family. The members of this large family of animal lectins interact with other proteins; the carbohydrate recognition domain of a galectin recognizes a β-galactoside conjugate on the interacting protein [10], and this interaction produces various biological effects [11]. Gal-3 is present both inside the cell and within the extracellular space [12]. It exists in the monomer form under soluble condition and forms pentamers when bound to the β-galactose of an interacting protein [13]. This characteristic allows Gal-3 to form bridges among cells through its multiple binding to β-galactose on different proteins. Gal-3 has been shown to regulate various cellular functions. For example, Gal-3 was found to promote tumor progression [14]; contribute to cell–extracellular matrix adhesion [15]; and promote inflammatory responses [16, 17]. The serum concentration of Gal-3 is increased in cancer patients, and Gal-3 enhances the homotypic aggregation of cancer cells and enables them to avoid anoikis by interacting with the cancer-associated mucin protein, MUC1 [18]. We recently reported that water maze training and contextual fear conditioning training both decrease Gal-3 expression in the rat hippocampus, and that memory performance is improved in Gal-3-knockout (KO) mice [19]. In the context of the present study, it is notable that the serum level of Gal-3 is increased in AD patients [20]. Together, these findings suggest that Gal-3 expression is negatively associated with memory function under both physiological and pathological conditions. However, although Gal-3 was reported to enhance cancer cell aggregation [18], it was previously unknown whether Gal-3 affects protein aggregation in the brain and/or whether Gal-3 plays a role in the pathology of AD. Here, we used APP/PS1 mice and brain tissues from AD patients to examine the role and action mechanism of Gal-3 in Aβ aggregation and amyloid plaque formation.

## Results

### Intra-hippocampal Aβ injection causes Aβ oligomerization

**Fig. 1:**
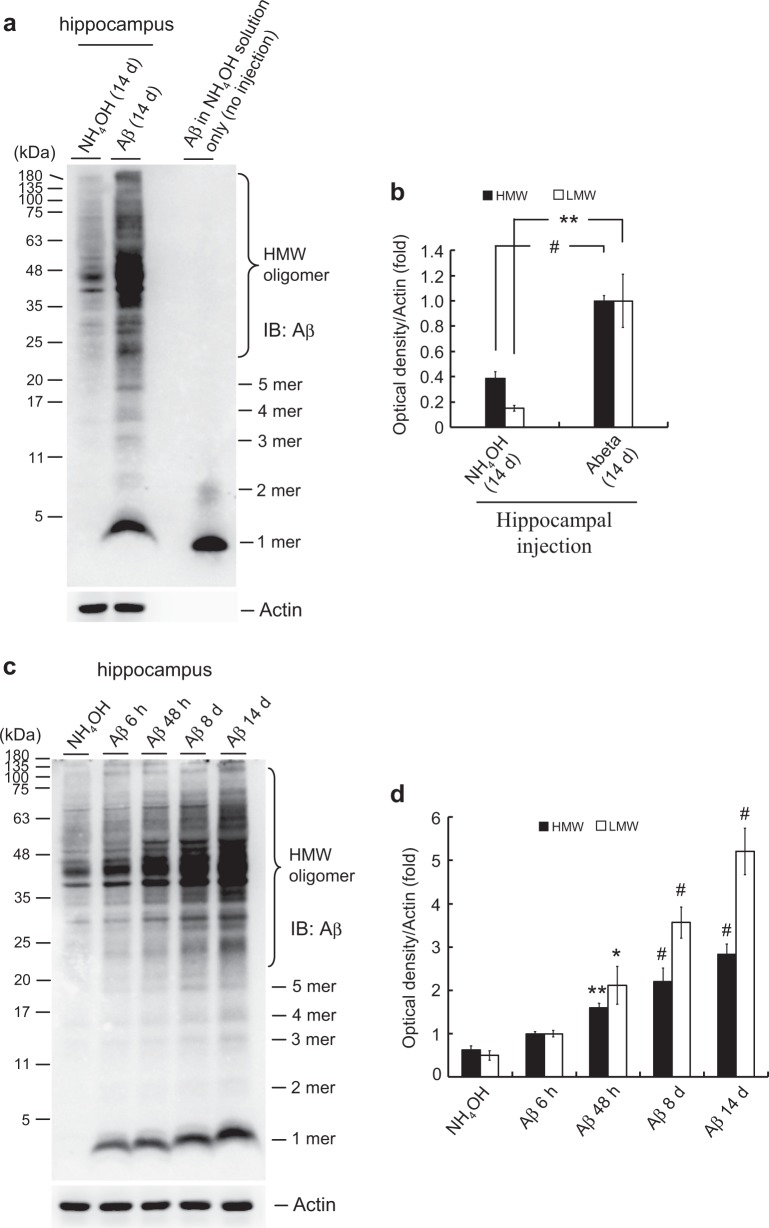
Intra-hippocampal Aβ injection causes Aβ oligomerization in mice. **a** Naïve adult mice (3 months old) received a single injection of 1% NH_4_OH or Aβ (14 μg) to their hippocampal CA1 area. Fourteen days later, dorsal hippocampal tissues were dissected and subjected to Western blot analysis of Aβ oligomerization (left two lanes). In another group, Aβ (0.2 μg) was dissolved in 1% NH_4_OH and directly loaded to a gel for examination of Aβ oligomerization (far right lane). Aβ oligomerization is presented as high molecular weight (HMW) oligomers (>23 kDa) and low-molecular weight (LMW) oligomers (<23 kDa). **b** Quantification of the results for HMW and LMW Aβ oligomerization [*n* = 4 per group; *t*(1,6) = 8.91, *P* < 0.001 for HMW and *t*(1,6) = 4.0, *P* < 0.01 for LMW]. **c** Mice (3 months old) received injections of 1% NH_4_OH or Aβ (14 μg) to the CA1 area and were sacrificed at different time points (6 h, 48 h, 8 days, and 14 days) postinjection. Dissected dorsal hippocampal tissues were subjected to Western blot analysis of Aβ oligomerization. **d** Quantified results for HMW and LMW Aβ oligomerization [*n* = 4 per group; *F*(4,15) = 22.71, *P* < 0.001 for HMW and *F*(4,15) = 29.71, *P* < 0.001 for LMW]. Data are expressed as mean ± SEM. Statistical significances of various comparisons are shown in the figure. *P < 0.05, **P < 0.01, ^#^*P* < 0.001 compared with the corresponding control group

We first examined whether exogenous Aβ injection produces Aβ oligomers in the hippocampus. Mice received intra-hippocampal NH_4_OH or Aβ injection and were sacrificed 14 days later. Their hippocampal tissues were dissected and subjected to Western blot analysis of Aβ oligomerization. Aβ oligomerization was assessed based on the presence of high-molecular weight (HMW) oligomers (>23 kDa) and low-molecular weight (LMW) oligomers (<23 kDa) [6, 21]. Results revealed that intra-hippocampal Aβ injection produced a significant amount of oligomerized Aβ in the hippocampus, whereas Aβ in the NH_4_OH solution (without intra-hippocampal injection) exhibited formation of only 2-mer Aβ oligomers (Fig. 1a, b). These results demonstrated that the Aβ oligomers seen following exogenous Aβ injection were formed in the brain rather than being formed in the solution per se.

Next, we examined the level of Aβ oligomerization over time after intra-hippocampal Aβ injection. Results indicated that Aβ oligomerization exhibited a time-dependent increase in the mouse hippocampus following Aβ injection. This effect was first apparent at 48 h postinjection and it was most significant at 14 days postinjection (Fig. 1c, d). The same results were also found in the rat hippocampus (Supplementary Fig. S1).

### Aβ oligomerization is reduced in Gal-3 KO mice injected with Aβ

**Fig. 2:**
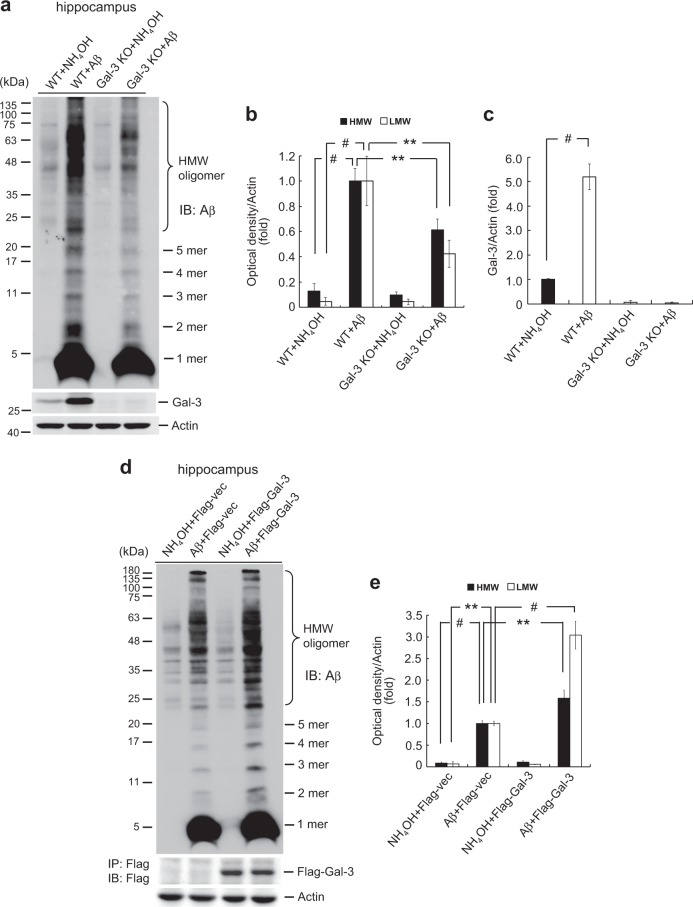
Aβ oligomerization is reduced in Gal-3 KO mice injected with Aβ. **a** WT and Gal-3 KO mice (3 months old) were injected with 1% NH_4_OH or Aβ (5 μg) in the CA1 area. Animals were sacrificed 48 h after injection and dissected hippocampal tissues were subjected to Western blot analysis of Aβ oligomerization and Gal-3 expression. **b** Quantified results of HMW and LMW Aβ oligomerization [*n* = 4 per group; *F*(3,12) = 149.28, *P* < 0.001 for HMW; *q* = 26.22, *P* < 0.001 for the WT+NH_4_OH group versus the WT+Aβ group and *q* = 13.78, *P* < 0.001 for the WT+Aβ group versus the Gal-3 KO+Aβ group; *F*(3,12) = 48.02 and *P* < 0.001 for LMW; *q* = 14.31, *P* < 0.001 for the WT+NH_4_OH group versus the WT+Aβ group and *q* = 6.74, *P* < 0.001 for the WT+Aβ group versus the Gal-3 KO+Aβ group]. **c** Quantified results for Gal-3 expression in the same mouse groups [*F*(3,12) = 83.43, *P* < 0.001; *q* = 15.63, *P* < 0.001 for the WT+NH_4_OH group versus the WT+Aβ group]. **d** Mice (3 months old) received the following intra-hippocampal injections: NH_4_OH (1%) injection and Flag-vector plasmid (0.4 μg) transfection; Aβ (5 μg) injection and Flag-vector plasmid transfection; NH_4_OH injection and Flag-Gal-3 plasmid transfection; and Aβ injection and Flag-Gal-3 plasmid transfection. The two injections were given 2 h apart and animals were sacrificed 48 h after the injection of NH_4_OH or Aβ. Dissected hippocampal tissues were analyzed for Aβ oligomerization. Tissue lysates were also subjected to immunoprecipitation and immunoblotting with anti-Flag antibody to confirm transfection and expression of the plasmid. **e** Quantified results for HMW and LMW Aβ oligomerization [*n* = 4 per group; *F*(3,12) = 52.71, *P* < 0.001 for HMW; *q* = 9.09, *P* < 0.001 for the NH_4_OH+Flag-vector group versus the Aβ+Flag-vector group and *q* = 5.8, *P* < 0.01 for the Aβ+Flag-vector group versus the Aβ+Flag-Gal-3 group; *F*(3,12) = 72.63, *P* < 0.001 for LMW; *q* = 5.65, *P* < 0.01 for the NH_4_OH+Flag-vector group versus the Aβ+Flag-vector group and *q* = 12.38, *P* < 0.001 for the Aβ+Flag-vector group versus the Aβ+Flag-Gal-3 group]. Data are expressed as mean ± SEM. ^**^P < 0.01, ^#^*P* < 0.001

Given that Gal-3 was previously shown to enhance cancer cell aggregation [18], we examined whether Gal-3 might also facilitate Aβ aggregation. Wild type (WT) and Gal-3 KO mice received Aβ or NH_4_OH injection to their CA1 area and were sacrificed 48 h later. The dissected hippocampal tissues were subjected to Western blot analysis of Aβ oligomerization and Gal-3 expression. We observed a light background signal and some inter-individual differences in the control group. Acute Aβ injection dramatically increased both HMW and LMW Aβ oligomerization. The Aβ oligomerization level was also low in Gal-3 KO mice, which showed some inter-individual differences. Aβ oligomerization was significantly lower in Gal-3 KO mice injected with Aβ compared to WT mice injected with Aβ (Fig. 2a, b). Meanwhile, Aβ injection markedly increased Gal-3 expression (Fig. 2a, c).

Since Aβ oligomerization was reduced in Gal-3 KO mice injected with Aβ, we hypothesized that overexpression of Gal-3 would facilitate Aβ oligomerization. To test this hypothesis, mice received different combinations of Aβ (or NH_4_OH) and Flag-tagged plasmid injections. The injections were given 2 h apart and animals were sacrificed at 48 h after Aβ (or NH_4_OH) injection. Their hippocampal tissues were assessed for Aβ oligomerization. Light background signal was similarly observed with inter-individual variation. Aβ injection increased both HMW and LMW Aβ oligomerization, but Aβ oligomerization was further enhanced in mice receiving Aβ and Flag-Gal-3 transfection. Plasmid transfection and expression were confirmed by immunoprecipitation and immunoblotting with an anti-Flag antibody (Fig. 2d, e).

### Aβ oligomerization and Gal-3 expression are age-dependently increased in APP/PS1 mice

**Fig. 3:**
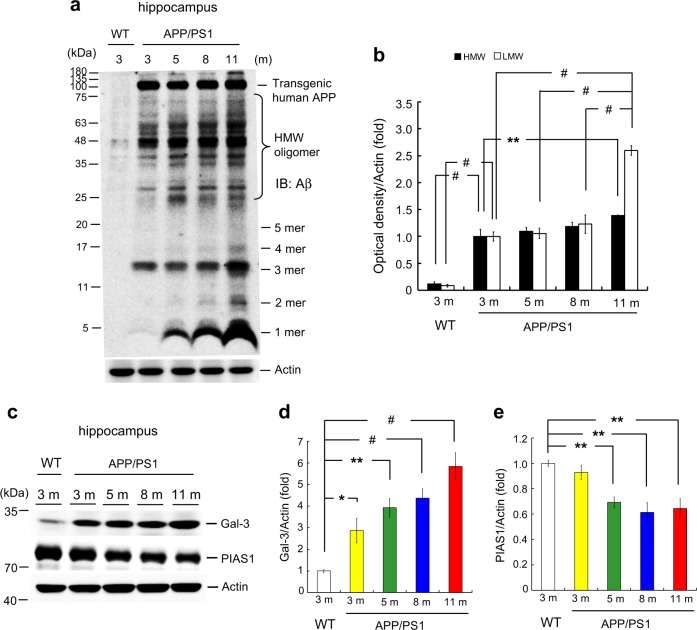
Aβ oligomerization and Gal-3 expression are age-dependently increased in APP/PS1 mice. **a** Wild-type (3 months old) and APP/PS1 mice of different ages (3, 5, 8, and 11 months) were sacrificed and dissected hippocampal tissues were subjected to Western blot analysis for endogenous Aβ oligomers. **b** Quantified results for HMW and LMW Aβ oligomerization [*n* = 4 per group; *F*(4,15) = 39.3, *P* < 0.001 for HMW and *F*(4,15) = 74.07, *P* < 0.001 for LMW]. Statistical significances for various comparisons are shown in the figure. **c** Western blot analysis showing the expression levels of Gal-3 and PIAS1 in hippocampal samples from the same batch of WT mice and APP/PS1 mice of different ages (*n* = 4 per group). **d** Quantified results for Gal-3 expression in WT and APP/PS1 mice [*F*(4,15) = 14.59, *P* < 0.001]. **e** Quantified results for PIAS1 expression in WT and APP/PS1 mice [*F*(4,15) = 5.86, *P* < 0.01] The letter “m” indicates month. Statistical significances of various comparisons are shown in the figure. Data are expressed as mean ± SEM. ^*^P < 0.05, ^**^P < 0.01, ^#^*P* < 0.001

The above results showed that Aβ oligomerization is observed in mice receiving intra-hippocampal Aβ injection. Here, we examined endogenous Aβ oligomerization in APP/PS1 mice of different ages. Results indicated that Aβ oligomerization was apparent in the hippocampus of APP/PS1 mice as early as 3 months of age compared with that in WT mice at 3-month old, and the level of Aβ oligomers increased age-dependently in the APP/PS1 mice (Fig. 3a, b).

Next, we examined endogenous Gal-3 expression of the same animals. Results revealed that Gal-3 expression was increased in APP/PS1 mice also in an age-dependent manner (Fig. 3c, d). To test the possibility that Gal-3 expression was increased simply due to aging, we examined Gal-3 expression in WT mice of different ages. The expression level of Gal-3 was found similar among WT mice of different ages (Supplementary Fig. S2a, b). Because Gal-3 promotes inflammatory responses [16, 17], we suspected that the age-dependent increases in Aβ oligomerization and Gal-3 expression observed among APP/PS1 mice might reflect an age-dependent increase in inflammation in these animals. Thus, we examined protein inhibitor of activated STAT1 (PIAS1), which exerts an important function in innate immune responses and anti-inflammation by negatively regulating STAT1 [22], and is expressed in the mouse and rat hippocampus [23, 24]. Indeed, we observed an age-dependent decrease of PIAS1 expression in the same APP/PS1 mice (Fig. 3c, e), but PIAS1 expression was similar among WT mice of different ages (Supplementary Fig. S2a, c).

### Endogenous Aβ oligomerization, Gal-3 expression, Iba1 and GFAP distribution, and amyloid plaques are reduced in APP/PS1;Gal-3^+/−^ mice

**Fig. 4:**
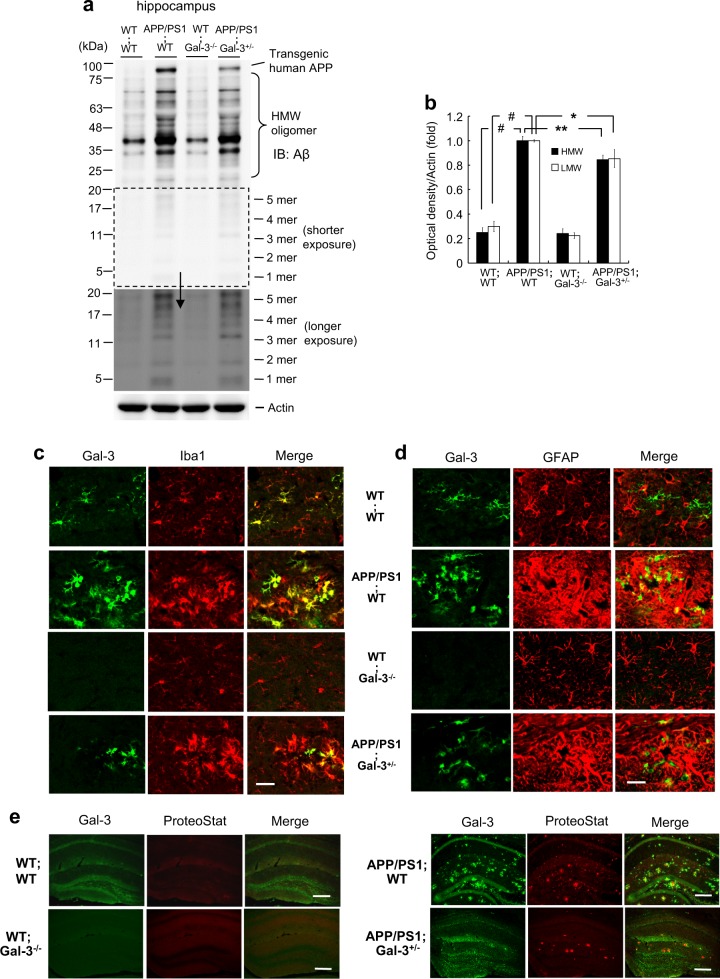
Endogenous Aβ oligomerization, Gal-3 expression, Iba1 and GFAP distribution, and amyloid plaque are reduced in APP/PS1;Gal-3^+/−^ mice. **a** Endogenous Aβ oligomerization was examined by Western blot analysis of samples obtained from 3-month-old WT;WT, APP/PS1;WT, WT;Gal-3^−/−^ and APP/PS1;Gal-3^+/−^ mice. **b** Quantified results for HMW and LMW Aβ oligomerization [*n* = 4 per group; *F*(3,12) = 110.36; *P* < 0.001 for HMW; *q* = 19.91, *P* < 0.001 for the WT;WT group versus the APP/PS1;WT group and *q* = 4.13, *P* = 0.01 for the APP/PS1;WT group versus the APP/PS1;Gal-3^+/−^ group; *F*(3,12) = 77.72, *P* < 0.001 for LMW; *q* = 15.87, *P* < 0.001 for the WT;WT group versus the APP/PS1;WT group and *q* = 3.32, *P* < 0.05 for the APP/PS1;WT group versus the APP/PS1;Gal-3^+/−^ group]. **c** Immunohistochemical analysis of Gal-3 and Iba1 and merged images for 7-month-old mice of the four genotypes. Scale bar, 25 μm. **d** Immunohistochemical analysis of Gal-3 and GFAP and merged image in 7-month-old mice of the four genotypes. Scale bar, 25 μm. **e** Immunohistochemical analysis showing the distribution of Gal-3 and ProteoStat in 11-month-old mice of the four genotypes. Scale bar, 200 μm. Data are expressed as mean ± SEM. ^*^P < 0.05, ^**^P < 0.01, ^#^*P* < 0.001

The above-described experiments involving intra-hippocampal Aβ injection and Gal-3 KO mice demonstrated that Gal-3 expression regulates Aβ oligomerization. Here, we used mice of different genotypes to further examine the role of Gal-3 in endogenous Aβ oligomerization. Adult WT;WT mice, APP/PS1;WT mice, WT;Gal-3^−/−^ mice and APP/PS1;Gal-3^+/−^ mice were sacrificed and dissected hippocampal tissues were subjected to Aβ oligomerization determination. A background signal and inter-individual differences in the WT;WT and WT;Gal-3^−/−^ mouse groups was observed. Significant Aβ oligomerization was observed in APP/PS1;WT mice, and this level was decreased by ~15% in APP/PS1;Gal-3^+/−^ mice (Fig. 4a, b).

Because Aβ oligomerization was decreased ~15% in APP/PS1;Gal-3^+/−^ mice compared with APP/PS1;WT mice and Gal-3 was found to activate microglia cells [25], we used immunohistochemical staining to examine whether Gal-3 expression and microglia distribution differed in hippocampal preparations of the two mouse groups. Ionized calcium-binding adaptor molecule 1 (Iba1) was used as a marker protein for microglia cells. Results showed that the expression levels of Gal-3 and Iba1 were much higher in APP/PS1;WT mice compared to WT;WT mice, and Gal-3 co-localized with Iba1 to a certain extent in APP/PS1;WT mice. WT;Gal-3^−/−^ mice lacked Gal-3 expression and showed a very low level of Iba1 expression. The expression level of Gal-3 was dramatically decreased in APP/PS1;Gal-3^+/−^ mice compared to APP/PS1;WT mice, and the expression of Iba1 was also reduced in APP/PS1;Gal-3^+/−^ mice relative to APP/PS1;WT mice (Fig. 4c). Besides, we also examined the role of astrocytes and their possible relationship with Gal-3. Glial fibrillary acidic protein (GFAP) was used as a marker protein for astrocytes. Results showed that the expression level of GFAP was much higher in APP/PS1;WT mice versus WT;WT mice, but Gal-3 only co-localized with GFAP in a small portion of these cells. The expression levels of Gal-3 and GFAP appeared to be reduced in APP/PS1;Gal-3^+/−^ mice compared to APP/PS1;WT mice (Fig. 4d). Further results revealed that Gal-3 and ProteoStat staining were both apparent in the hippocampus of APP/PS1;WT mice, and they co-localized to a certain extent. WT;WT mice and WT;Gal-3^−/−^ mice showed little staining, and the levels of Gal-3 and ProteoStat staining appeared lower in APP/PS1;Gal-3^+/−^ mice compared to APP/PS1;WT mice (Fig. 4e).

### Endogenous Gal-3 expression is decreased but memory performance is improved in APP/PS1;Gal-3^+/−^ mice

**Fig. 5:**
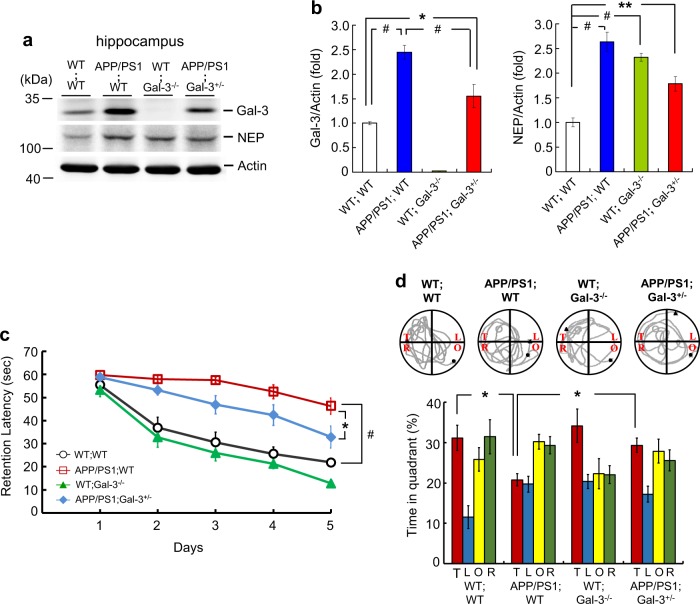
Endogenous Gal-3 expression is decreased but memory performance is improved in APP/PS1;Gal-3^+/−^ mice. **a** Endogenous expression levels of Gal-3 and NEP were examined by Western blot analysis in 3-month-old WT;WT, APP/PS1;WT, WT;Gal-3^−/−^ and APP/PS1;Gal-3^+/−^ mice. **b** Quantified results for Gal-3 and NEP expression [*n* = 4 per group; for Gal-3, *F*(3,12) = 53.47, *P* < 0.001; *q* = 10.43, *P* < 0.001 for the WT;WT group versus the APP/PS1;WT group; *q* = 6.46, *P* < 0.001 for the APP/PS1;WT group versus the APP/PS1;Gal-3^+/−^ group and *q* = 3.98, *P* < 0.05 for the WT;WT group versus the APP/PS1;Gal-3^+/−^ group; for NEP, *F*(3,12) = 27.76, *P* < 0.001; *q* = 12.04, *P* < 0.001 for the WT;WT group versus the APP/PS1;WT group; *q* = 9.72, *P* < 0.001 for the WT;WT group versus the WT;Gal-3^−/−^ group and *q* = 6.29, *P* < 0.01 for the WT;WT group versus the APP/PS1;Gal-3^+/−^ group]. **c** Acquisition performance obtained by assessing the water maze learning of 8-month-old mice of the four genotypes [*n* = 7 per group; *F*(3,24) = 28.72, *P* < 0.001; *q* = 9.33, *P* < 0.001 for the APP/PS1;WT group versus the WT;WT group and *q* = 3.59, *P* < 0.05 for the APP/PS1;Gal-3^+/−^ group versus the APP/PS1;WT group]. **d** Retention performance of the same mice described in (**c**) [*n* = 7 per group; *F*(3,24) = 4.76, *P* < 0.01; *q* = 3.94, *P* < 0.05 for the APP/PS1;WT group versus the WT;WT group; *q* = 3.25, *P* < 0.05 for the APP/PS1;Gal-3^+/−^ group versus the APP/PS1;WT group for the target quadrant]. Representative swim patterns from each group are also shown (upper panel). Data are expressed as mean ± SEM. ^*^P < 0.05, ^**^P < 0.01, ^#^*P* < 0.001

We next examined the expression level of Gal-3 in the animals addressed in Fig. 4a. Results showed that Gal-3 expression was consistently higher in APP/PS1;WT mice than in WT;WT mice, whereas it was decreased by about 40% in APP/PS1;Gal-3^+/−^ mice relative to APP/PS1;WT mice (Fig. 5a, b). Neprilysin (NEP) is an enzyme that mainly acts to degrade extracellular Aβ42 [26, 27]. A previous study noted accumulation of Aβ42 in the brains of NEP KO mice [28]. Thus, we examined NEP expression in our mouse groups. We found that NEP expression was higher in APP/PS1;WT mice, WT;Gal-3^−/−^ mice and APP/PS1;Gal-3^+/−^ mice compared with WT;WT mice (Fig. 5a, b).

We also examined the cognitive performance in these four genotypes of mice by adopting the water maze learning task. Results showed that APP/PS1;WT mice exhibited much slower acquisition than the WT;WT mice, while APP/PS1;Gal-3^+/−^ mice showed an acquisition performance intermediate between those of WT;WT and APP/PS1;WT mice (Fig. 5c). Similar results were found with the retention test (Fig. 5d). The swim speeds were similar among these four mouse groups (Supplementary Table 1).

### Galectin-3 associates with Aβ and interacts with Aβ

**Fig. 6:**
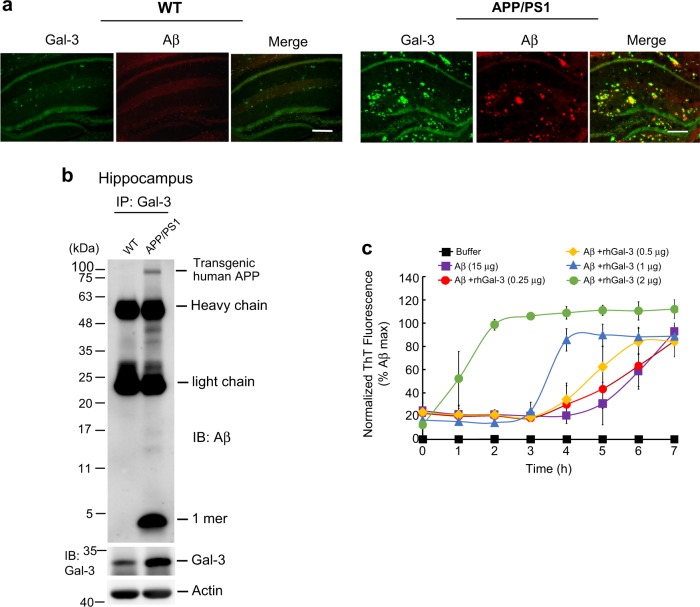
Galectin-3 associates with Aβ and interacts with Aβ. **a** Immunohistochemical analysis showing the distributions of endogenous Gal-3 and Aβ in hippocampal samples of WT (left panel) and APP/PS1 (right panel) mice at 11 months of age. Scale bar, 200 μm. **b** Co-IP experiment showing preferential association of Gal-3 with endogenous Aβ monomers in 8-month-old APP/PS1 mice. Experiments were performed in duplicate. **c** Different amounts of recombinant human Gal-3 protein (0.25, 0.5, 1, and 2 μg) were added to a solution containing Aβ42 peptide (15 μg), the mixture was subjected to a thioflavin-T assay, and fluorescence was measured hourly at different time points. The fluorescence plateaued at the 7-h time point. Experiments were performed in triplicate. The abbreviation “rh” indicates “recombinant human”. Data are expressed as mean ± SEM

The above-described results showed that Gal-3 mainly co-localizes with microglia cells and Aβ oligomerization largely takes place in the extracellular space. Here, we examined the possible co-localization of Gal-3 with endogenous Aβ in the hippocampus of WT and APP/PS1 mice. Immunohistochemical analyses revealed that WT mice exhibited low-level Gal-3 expression and almost no Aβ staining, whereas APP/PS1 mice show higher-level and overlapping expression of both Gal-3 and Aβ (Fig. 6a). Given this, we further examined the relationship between Gal-3 and Aβ by performing a co-immunoprecipitation experiment. Results revealed that Gal-3 was preferentially associated with endogenous Aβ monomers, and Gal-3 expression was consistently increased in the hippocampus of APP/PS1 mice (Fig. 6b). Next, we examined whether there is a direct interaction between Gal-3 and Aβ. Synthetic Aβ peptide was mixed with different amounts of human recombinant Gal-3 protein and incubated for 18 h. A thioflavin-T assay was performed and the fluorescence intensity was measured hourly. We found that incubation of Aβ alone yielded fluorescence as the incubation time increased, and Gal-3 dose-dependently enhanced the observed fluorescence (Fig. 6c).

Because Gal-3 co-localized with Iba1 in APP/PS1 mice and Gal-3 was reported to activate microglia cells through Toll-like receptor 4 (TLR4) [25], we examined the Gal-3 target protein on microglia in the context of AD. Triggering receptor expressed on myeloid cells-2 (TREM2) is a microglial protein for which a genetic variant has been associated with the risk of AD [29]. Here, we used co-immunoprecipitation experiments to examine whether Gal-3 is associated with TREM2. Results revealed that Gal-3 associated with TREM2 and TLR4 (positive control) in the in vitro assay (Supplementary Fig. S3a). By adopting a TREM2 siRNA and BV-2 cells, we further found that TREM2 mediates the effect of Gal-3 on microglia activation (Supplementary Fig. S3b).

### NEP expression is increased and integrin signaling is enhanced in Gal-3 KO mice

**Fig. 7:**
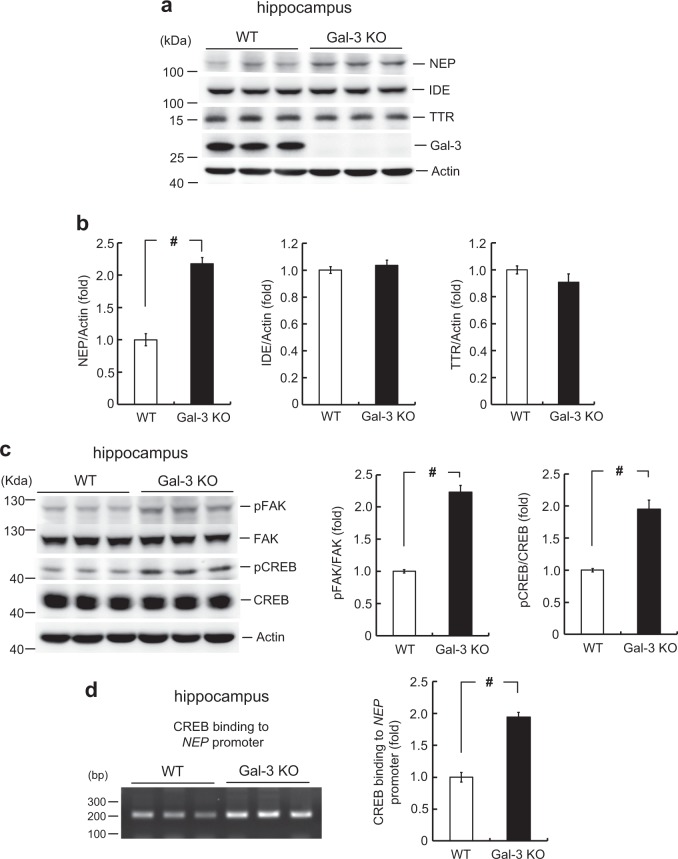
Neprilysin (NEP) expression is increased and integrin signaling is enhanced in Gal-3 KO mice. **a** Dissected hippocampi of naïve WT and Gal-3 KO mice (3-month-old) were subjected to various Western blot assays. Representative gel patterns obtained for NEP, IDE, TTR, and Gal-3 are shown. **b** Quantified results for NEP expression [*t*(1,8) = 8.61, *P* < 0.001; left panel], IDE expression [*t*(1,8) = 0.72, *P* > 0.05; middle panel] and TTR expression [*t*(1,8) = 1.32, *P* > 0.05; right panel]. **c** Representative gel patterns obtained for pFAK, FAK, pCREB, and CREB, and their quantified results [*t*(1,8) = 11.14, *P* < 0.001 for pFAK/FAK; *t*(1,8) = 0.57, *P* > 0.05 for FAK/Actin; *t*(1,8) = 6.72, *P* < 0.001 for pCREB/CREB and *t*(1,8) = 0.67, *P* > 0.05 for CREB/Actin]. **d** ChIP assay examining the binding of endogenous CREB to the *NEP* promoter in WT and Gal-3 KO mice, and the quantified results [*t*(1,8) = 9.06, *P* < 0.001]. Data are expressed as mean ± SEM. ^#^*P* < 0.001

The above-described experiments showed that Aβ oligomerization was decreased in Gal-3 KO mice treated with Aβ compared to similarly treated WT mice, and that overexpression of Gal-3 increased exogenous Aβ-induced Aβ oligomerization. Here, we examined the underlying mechanism of Gal-3 action. Besides NEP, a few other enzymes are known to degrade Aβ or prevent Aβ accumulation [30]. For example, insulin-degrading enzyme (IDE) degrades both Aβ40 and Aβ42 peptides and cleaves Aβ at multiple sites [31, 32]. Transthyretin (TTR) is known to prevent the aggregation of LMW Aβ to generate HMW Aβ [33]. As these enzymes play important roles in the clearance of endogenous Aβ, we examined whether their expression levels are altered in Gal-3 KO mice. Results revealed that the basal expression level of NEP was higher in Gal-3 KO mice than WT mice (approximately 2.2-fold), the expression levels of IDE and TTR were similar between WT and Gal-3 KO mice (Fig. 7a, b).

Gal-3 is suggested to bind the β-galactose conjugate on integrin [30, 34]. Notably, integrin was shown to facilitate memory function in *Drosophila* and mice [35, 36], and we recently demonstrated that Gal-3 impairs memory formation through inhibition of integrinα3-mediated signaling [19]. Based on these findings, we herein examined whether integrin signaling is increased in Gal-3 KO mice. We measured the phosphorylation level of focal adhesion kinase (FAK), which has been shown to mediate integrin signaling [37]. We also measured the phosphorylation level of cyclic AMP-responsive element binding protein (CREB), whose phosphorylation is a downstream event of FAK phosphorylation which is associated with neuronal plasticity [38]. We found that the phosphorylation levels of FAK and CREB were increased in Gal-3 KO mice (Fig. 7c). To examine whether this signaling pathway could regulate *NEP* gene expression, we performed a chromatin immunoprecipitation assay. We found that the binding of endogenous CREB to the *NEP* promoter was higher (approximately twofold) in the hippocampus of Gal-3 KO mice (Fig. 7d).

### Aβ oligomerization and Gal-3 expression are increased in AD patients

**Fig. 8:**
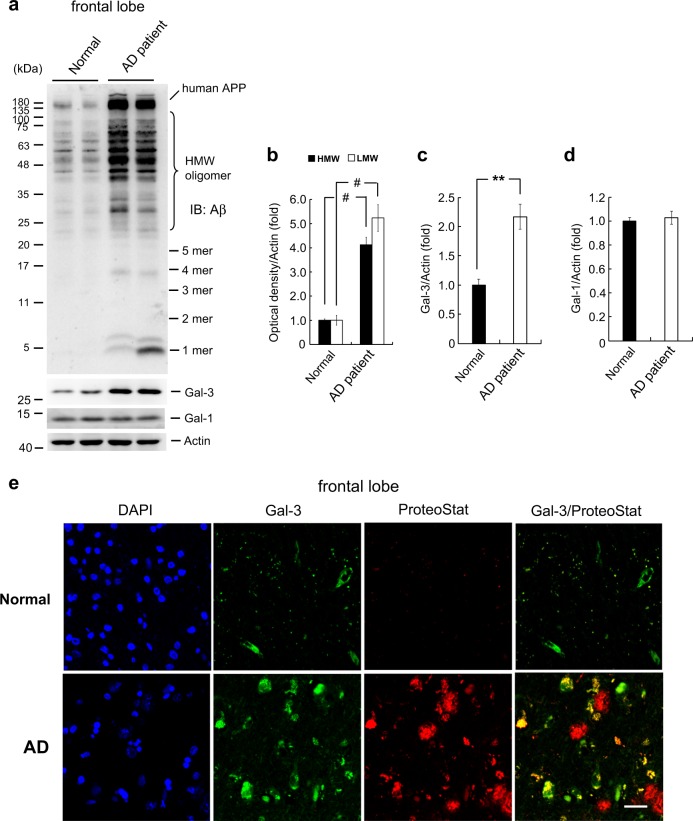
Aβ oligomerization and Gal-3 expression are increased in AD patients. The frontal lobe lysates of normal subjects and AD patients were subjected to Western blot analysis for Aβ oligomerization and the expression of Gal-3 and Gal-1 (*n* = 4 per group). **b**–**d** Quantified results for (**b**) HMW [*t*(1,6) = 9.72, *P* < 0.001] and LMW [*t*(1,6) = 7.21, *P* < 0.001] Aβ oligomerization, **c** Gal-3 expression [*t*(1,6) = 4.92, *P* < 0.01] and **d** Gal-1 expression [*t*(1,6) = 0.43, *P* > 0.05]. **e** Immunohistochemical analysis showing the distributions of Gal-3 and ProteoStat staining in normal subjects and AD patients (*n* = 3 per group). Scale bar, 25 μm. DAPI was used to stain nuclei. Data are expressed as mean ± SEM. ^**^P < 0.01, ^#^*P* < 0.001

The above results showed that Gal-3 expression is increased in APP/PS1 mice and Gal-3 promotes Aβ oligomerization, but it remained unclear whether Gal-3 contributes to the pathology of AD. To address this issue, we examined Gal-3 expression and Aβ oligomerization in the frontal lobes of normal subjects and AD patients. We observed a significantly higher amount of Aβ oligomerization in AD patients than in normal subjects (Fig. 8a, b). The expression level of Gal-3 was also higher in AD patients (Fig. 8a, c). To examine the specificity of the relationship between Gal-3 and Aβ oligomerization, we used the same tissue lysates to determine the expression level of Gal-1. Results indicated that Gal-1 expression was similar between normal subjects and AD patients (Fig. 8a, d).

We also examined Gal-3 expression and ProteoStat staining in the frontal lobes of normal subjects and AD patients. Immunohistochemical results revealed that Gal-3 co-localized with smaller plaques, but not large plaques, in AD patients, whereas little staining for Gal-3 or ProteoStat was observed in normal subjects (Fig. 8e). Finally, we found that the serum Gal-3 level increased with the severity of memory loss in different stages of AD patients (Supplementary Fig. S4).

## Discussion

In the present study, we show that a single injection of Aβ to the mouse hippocampus causes Aβ oligomerization within a short period (48 h to 14 days). This likely reflects that a high concentration of Aβ (about 1 mM) was administered to the brain, and only some of it could be physiologically degraded. The remaining Aβ thus yielded apparent oligomerization within a short time. In another study, immunohistochemical analysis revealed that a single injection of Aβ induces Aβ oligomer deposits in the mouse hippocampus 5 min after injection [39]. In the present study, we observed very low levels of Aβ oligomerization in the control groups of some experiments. This may also reflect that the injection contained a high concentration of Aβ, and thus triggered a significant amount of oligomerization. In contrast, the oligomerization level for the control groups was relatively low.

Our results indicate that Gal-3 expression is induced by Aβ stimulation and increases with age in APP/PS1 mice. This primarily occurs in microglia cells. Gal-3 promotes Aβ aggregation by directly interacting with Aβ and decreasing the degradation of Aβ. Together, our results suggest that the ability of Aβ to induce Gal-3 expression in microglia mediates the inflammatory response. These results are consistent with a previous report that Gal-3 mediates α-synuclein-induced microglia activation [40]. Our observation that Gal-3 facilitates Aβ oligomerization is also consistent with a previous report that Gal-3 enhances cancer cell aggregation [18], but contrasts with a study showing that Gal-3 reduces Aβ neurotoxicity [41]. This discrepancy is probably due to differences between the in vivo and in vitro settings. Further studies are warranted to examine whether Gal-3 facilitates the aggregation of additional proteins associated with other neurodegenerative diseases.

Our in vivo co-IP results indicated that Gal-3 mainly associates with Aβ monomers. Gal-3 is known to recognize and interact with β-galactose conjugates on other proteins [10]. O-glycosylation can take place at Tyr-10 of the Aβ1-19 peptide in human cerebrospinal fluid and is increased in AD patients [42]. It is possible that Gal-3 binds to the glyco-conjugate on Aβ peptide and further promotes Aβ aggregation. However, we found that Gal-3 also directly interacts with Aβ in the in vitro setting, where glycosylation does not take place. Thus, we speculate that Gal-3 interacted with the β-sheet structure of Aβ in vitro, and that this interaction may also occur under physiological conditions. Our observation that Gal-3 preferentially associates with Aβ monomers seems to explain why the distribution of Gal-3 does not completely overlap with amyloid plaque staining in APP/PS1 mice and AD patients, given that amyloid plaque largely consists of Aβ fibrils.

Our experiments revealed that exogenous Aβ-induced Aβ oligomerization is reduced in Gal-3 KO mice. In examining the mechanism underlying this phenomenon, we found that the expression level of endogenous NEP is about twofold higher in Gal-3 KO mice compared to WT mice. Because NEP mainly degrades extracellular Aβ42 [27], our results suggest that in Gal-3 KO mice, the generated Aβ is efficiently degraded before it forms oligomers. Further, the lack of Gal-3 in these mice would block the interaction between Gal-3 and Aβ, and consequently reduce the formation of Aβ oligomers. Moreover, NEP expression is also increased in APP/PS1;WT mice and APP/PS1;Gal-3^+/−^ mice. This likely reflects that the Aβ level is elevated in these animals, such that more NEP is generated to efficiently degrade Aβ as an endogenous protection mechanism.

While further examining the role of Gal-3 in Aβ oligomerization, we found that the levels of Aβ oligomerization and amyloid plaque are both reduced in APP/PS1;Gal-3 heterozygous mice compared to APP/PS1;WT mice. This observation supports our proposal that Gal-3 facilitates Aβ oligomerization. The functional significance of the reduced Aβ oligomerization in APP/PS1;Gal-3 heterozygous mice was exemplified by their performance in the water maze, where APP/PS1;Gal-3 heterozygous mice showed better acquisition and retention performance than APP/PS1;WT. Although Aβ oligomerization was only ~15% lower in APP/PS1;Gal-3^+/−^ mice compared to APP/PS1;WT mice at 3 months of age, there was a clear between-group difference in amyloid plaque at 11 months of age. It is possible that more Aβ oligomers and Aβ fibrils are degraded in APP/PS1;Gal-3^+/−^ mice at an earlier stage, before the later formation of amyloid plaques. Several enzymes are known to degrade Aβ oligomers or Aβ fibrils [43]. Thus, it could be worthwhile to examine whether the expression and/or activity of these enzymes are increased in APP/PS1;Gal-3^+/−^ mice. Moreover, although Gal-3 expression was decreased by about 40% in APP/PS1;Gal-3^+/−^ mice compared to APP/PS1;WT mice at 3 months of age, this difference was much more pronounced at 11 months of age. This likely reflects that the decreased Gal-3 expression in adult APP/PS1;Gal-3^+/−^ mice yielded less microglia activation and inflammation during the aging process, which further reduced Gal-3 expression. This explanation is supported by our observation that the distribution of microglia is reduced in APP/PS1;Gal-3^+/−^ mice compared to APP/PS1;WT mice at 7 months of age. The distribution of astrocytes is also lower in APP/PS1;Gal-3^+/−^ mice compared to APP/PS1;WT mice, suggesting that astrocytes may also play a role in Gal-3-mediated inflammation. Further, when we examined Aβ oligomerization in APP/PS1;Gal-3^−/−^ mice, we were surprised to find that Aβ oligomerization is increased in adult APP/PS1;Gal-3^−/−^ mice versus APP/PS1;WT mice (Supplementary Fig. S5). This may reflect that compensatory mechanisms have been activated by Gal-3 deficiency in these animals.

Lastly, we herein show that Gal-3 expression is significantly increased in the frontal lobe of AD patients in parallel with enhanced Aβ oligomerization, and that the serum level of Gal-3 increases with the severity of memory loss in AD patients. The latter result is consistent with a previous report that the serum level of Gal-3 is elevated in AD patients [20]. Accumulating evidence demonstrates that Aβ oligomers play important and pathogenic roles in AD [44, 45]. Thus, compounds that work against the aggregation of Aβ oligomers are considered to have therapeutic potential against AD [46]. In this context, the present work further suggests that agents capable of downregulating Gal-3 expression could also be ideal candidates as novel therapeutic agents to combat AD.

## Materials and methods

### Animals

Adult male Sprague–Dawley rats (250–300 g), adult male and female C57BL/6J WT mice, male and female Gal-3 KO mice, male and female APP/PS1 transgenic mice were used in this study. The APP/PS1 mice (strain name: B6.Cg-Tg (APPswe,PSEN1dE9)85Dbo/Mmjax, stock number: 005864), Gal-3 KO mice (stock number: 006338) and WT mice (strain name: C57BL/6J, stock number: 000664) were purchased from Jackson Laboratory (Bar Harbor, ME, USA). Animals were bred at the Animal Facility of the Institute of Biomedical Sciences (IBMS), Academia Sinica, Taiwan. They were housed and maintained on a 12/12 h light/dark cycle (light on at 8:00 A.M.) with food and water continuously available. Experimental procedures followed the Guidelines of Animal Use and Care of the National Institute of Health (NIH) and were approved by the Animal Committee of IBMS, Academia Sinica.

### Synthetic Aβ peptide

Synthetic human Aβ (1–42) peptide was purchased from GenScript (Piscataway, NJ). Aβ was dissolved in 1% NH_4_OH before injection.

### Plasmid DNA construction

For construction of the Flag-tagged Gal-3 plasmid, full-length *LGALS3* was cloned by amplifying the mouse hippocampal cDNA with primers 5′-ATCGGGATCCATGGCAGACAGCTTTTC-3′ (forward) and 5′-CGATAAGCTTTTAGATCATGGCGTGGTTAG-3′ (reverse). The PCR product was sub-cloned into the *BamHI* and *HindIII* sites of the mammalian expression vector pCMV-Tag2B (Invitrogen, Carlsbad, CA).

### Intra-hippocampal Aβ peptide infusion and plasmid DNA transfection

Animals were anesthetized with pentobarbital (40 mg/kg, i.p.) and subjected to stereotaxic surgery without cannulation. Aβ_1–42_ (20 μg/μl) or Flag-Gal-3 plasmid (1.5 μg/μl) was directly injected to the CA1 area of anesthetized animals at a rate of 0.1 μl/min. A total of 0.7 μl was injected to each side of CA1 area in rats and 0.25 μl in mice. Transient Flag-Gal-3 plasmid DNA transfection was conducted using the nonviral transfection agent polyethyleneimine (PEI) because we have previously demonstrated that PEI does not produce toxicity to hippocampal neurons [47]. The procedures were adopted from that described previously [48]. Briefly, plasmid DNA was diluted in 5% glucose to a stock concentration of 2.77 μg/μl. Branched PEI of 25kDa (Sigma, St. Louis, MO) was diluted to 0.1 M concentration in 5% glucose and added to the DNA solution. Immediately before injection, 0.1 M PEI was added to reach a ratio of PEI nitrogen per DNA phosphate equals to 10. The mixture was subjected to vortex for 30 s and allowed to equilibrate for 15 min. The injection needle was left in place for 5 min to limit the diffusion of injected agent. Animals were sacrificed 48 h after Flag-Gal-3 plasmid DNA transfection or at different periods after Aβ injection. For the experiment that requires two injections, Aβ was injected 2 h after Flag-Gal-3 plasmid transfection and mice were sacrificed 48 h after Aβ injection. Their hippocampal tissue was dissected out and frozen at −80 °C until biochemical assays.

### Hippocampal cell lysate preparation

The hippocampal tissue was lysed by brief sonication in lysis buffer containing 50 mM Tris-HCl (pH 7.4), 150 mM NaCl, 2 mM EDTA and 1% IGEPAL CA-630. One tablet of protease inhibitor cocktail (cOmplete^™^ ULTRA Tablets, Mini, EDTA-free, EASYpack, Roche, Mannheim, Germany) and one tablet of phosphatase inhibitor (PhosSTOP, Roche) were added to each 10 ml of the lysis buffer before sonication. The homogenate was centrifuged at 14,000× rpm for 10 min at 4 °C. The supernatant was collected and stored at −80 °C for further analysis.

### Detection of Aβ oligomerization

Protein concentration of lysate was quantified by Bio-Rad Protein Assay Dye Reagent Concentrate (Bio-Rad, Hercules, CA). The clarified lysate (16 μg) was mixed with 5× protein loading buffer (GeneMark) before boiling at 95 °C for 10 min and were subjected to 13% Tricine sodium dodecyl sulfate polyacrylamide gel electrophoresis (SDS-PAGE) followed by transferring onto the polyvinylidene difluoride (PVDF) membrane (Millipore, Bedford, MA). Before blocking with 5% skim milk, membrane was submerged in boiling phosphate-buffered saline (PBS) for 6 min. Immunoblot was conducted using the mouse anti-human amyloid-beta 6E10 antibody (1:3000, BioLegend, San Diego, CA). The secondary antibody used was horseradish peroxidase (HRP)-conjugated goat anti-mouse IgG antibody (1:6500, Jackson ImmunoResearch, West Grove, PA). Membrane was developed by reacting with chemiluminescence HRP substrate (Millipore) and exposed to the LAS-3000 image system (Fujifilm, Tokyo, Japan) for visualization of protein bands. The protein bands were quantified using the NIH Image J Software.

### Western blot

Cell lysates were resolved by 8–12% SDS-PAGE and transferred onto the PVDF membrane. Immunoblotting was carried out using the following antibodies: rat anti-Gal-3 (1:5000, R&D systems), goat anti-Gal-1 (1:5000, R&D systems), rabbit anit-PIAS1 (1:3000, Epitomics, Burlingame, CA), mouse anti-CD10/NEP (1:500, Santa Cruz Biotechnology, Dallas, TX), mouse anti-prealbumin/transthyretin (1:500, Santa Cruz Biotechnology), rabbit anti-insulin degrading enzyme (1:3000, Abcam, Cambridge, UK), rabbit anti-FAK (1:2000, Abcam), rabbit anti-pFAK (phospho-Y397, 1:2000, Abcam), rabbit anti-CREB (1:2000, Cell Signaling, Danvers, MA), rabbit anti-pCREB (phospho-S133, 1:2000, Cell Signaling), rat anti-TREM2 (1:3000, Millipore, Bedford, MA), mouse anti-TLR4 (1:500, Santa Cruz Biotechnology), rabbit anti-CD40 (1:2000, Abcam), mouse anti-actin (1:150000, Millipore), and mouse anti-Flag M2 (1:10000, Sigma-Aldrich) antibodies. The secondary antibodies used were HRP-conjugated goat anti-rabbit, goat anti-mouse, goat anti-rat and donkey anti-goat IgG antibodies (1:6000, Jackson ImmunoResearch). Membrane was developed by reacting with chemiluminescence HRP substrate and exposed to the LAS-3000 image system (Fujifilm) for visualization of protein bands. The protein bands were quantified using the NIH Image J Software.

### Thioflavin-T Αβ aggregation assay

Aβ42 aggregation was performed according to the protocol of SensoLyte® Thioflavin T beta-amyloid (1–42) aggregation kit from AnaSpec (Catalog No. AS-72214, Fremont, CA). Ten microliters of 2 mM thioflavin-T working solution were mixed with 60 μl (15 μg) Aβ42 peptide solution (0.25 mg in 1 ml cold assay buffer) in each microplate well, then 1.25 μl (0.25 μg), 2.5 μl (0.5 μg), 5 μl (1 μg) and 10 μl (2 μg) of recombinant human Gal-3 protein (200 µg/ml, Catalog no. 8259-GA-050, R&D) was added to each well and bring the total volume of all samples to 100 μl with assay buffer. One hundred microliters of the assay buffer were added as a blank. Fluorescence intensity was measured immediately at 37 °C for 18 h. The samples were read in a Gemini EM fluorescence microplate reader system (Device, Sunnyvale, CA) using a filter with the excitation wavelength set at 440 nm and emission wavelength set at 480 nm.

### Co-immunoprecipitation (Co-IP)

The hippocampal tissues from WT and APP/PS1 mice were lysed in RIPA buffer (50 mM Tris-HCl [pH 7.4], 150 mM NaCl, 1% IGEPAL CA-630, 1 mM EDTA and 1 mM EGTA) with cocktail of protease and phosphatase inhibitor (Roche). The clarified lysate (0.5 mg) was immunoprecipitated with 3 μl of anti-Gal-3 antibody (R&D systems) at 4 °C for overnight. Twenty microlitre (10% slurry) of the Mag Sepharose Xtra beads (GE Healthcare, Pittsburgh, PA) was added to the IP reaction product for overnight to catch the immune complex. The immune complex on beads was washed three times with PBS and boiled in sample buffer at 95 °C for 10 min. The product was then subjected to SDS-PAGE followed by transferring onto the PVDF membrane and immunoblotted with anti-Aβ 6E10 antibody. To confirm Flag-tagged plasmid transfection and expression in the CA1 area, the CA1 tissue was lysed in RIPA buffer and the clarified lysate (0.5 mg) was immunoprecipitated with 3 μl of anti-Flag-M2 antibody at 4 °C for overnight. Other procedures were the same as that described above except that the membrane was immunoblotted with the Flag-M2 antibody.

### Co-IP for Gal-3 protein-protein interaction

The recombinant human Gal-3 protein (0.6 μg, 0.2 μg/μl in PBS, catalog No. 8259-GA-050, R&D systems) was mixed with recombinant human TLR4 protein (0.6 μg, 0.2 μg/μl in PBS, catalog No. 1478-TR-050, R&D systems) and recombinant human TREM2 protein (0.6 μg, 0.2 μg/μl in PBS, catalog No. 9256-T2-050, R&D systems), respectively, in RIPA buffer. The mixture was immunoprecipitated with 3 μl of rat anti-Gal-3 antibody (0.2 μg/μl in PBS, catalog No. MAB1197, R&D systems) at 4 °C for overnight. Rat IgG (0.6 μg, 0.2 μg/μl in PBS, catalog No. MAB006, R&D systems) was used as the control group. The protein G Mag Sepharose Xtra beads (15 μl, 50% slurry, GE Healthcare, Chicago, IL) were added to the IP reaction product to catch the immune complex at 4 °C for overnight. The immune complex on beads were washed three times with 1× PBS before boiling at 95 °C for 10 min and subjected to 12% SDS-PAGE followed by transferring onto the PVDF membrane (Millipore).

### Chromatin immunoprecipitation (ChIP) assay

ChIP assay was performed according to the protocol of ChIP assay kit (Catalog No. 17-10085) from Millipore. The mouse hippocampus tissue was washed with 1× ice-cold PBS and fixed with 1% formaldehyde by adding formaldehyde to the ice-cold PBS for 10 min. After adding glycine to quench the unreacted formaldehyde, tissue was homogenized and resuspended in cell lysis buffer plus protease inhibitor cocktail II, then changed to nuclear lysis buffer plus protease inhibitor cocktail II for sonication. The chromatin was immunoprecipitated using rabbit anti-CREB antibody (Cell Signaling, Danvers, MA). DNA purified from the immunoprecipitated samples was subjected to PCR reaction. The forward primer used for the *NEP* promoter is: 5′-GTTGCAGTATCACAATGTG-3′ (nucleotide −3034 to −3016) and the reverse primer is: 5′-GACTAAACAGAAACTCCAC-3′ (nucleotide −2841 to −2823). The PCR product of the *NEP* promoter is 212 bps in length. The PCR product was separated by 2% agarose gel electrophoresis.

### Genotyping

APP/PS1 transgenic mice were mated with WT mice to maintain the APP/PS1 strain and the mating system was APP/PS1^(+/−)^ × WT. All offspring were subjected to APP/PS1 genotyping and 50% of the offspring were APP/PS1 heterozygous. For Gal-3, male Gal-3 KO mice were mated with female Gal-3 KO mice to maintain the Gal-3 KO strain and the mating system was Gal-3^(−/−)^ × Gal-3^(−/−)^. All offspring were subjected to Gal-3 genotyping and 100% of offspring were Gal-3 KO homozygous. The APP/PS1^(+/−)^;Gal-3 ^(+/−)^ mice were generated from APP/PS1 and Gal-3 KO mice. The mating system was APP/PS1^(+/−)^ × Gal-3^(−/−)^. All offspring were subjected to APP/PS1 and Gal-3 genotyping and 50% of the offspring were APP/PS1^(+/−)^;Gal-3^(+/−)^ mice. The APP/PS1^(+/−)^;Gal-3^(−/−)^ mice were generated from APP/PS1^(+/−)^;Gal-3^(+/−)^ mice and Gal-3^(−/−)^ mice. The mating system was the APP/PS1^(+/−)^;Gal-3^(+/−)^ genotype mice that further mated with the Gal-3^(−/−)^ mice. All offspring were subjected to APP/PS1 and Gal-3 genotyping and 25% of the offspring were the APP/PS1^(+/−)^;Gal-3^(−/−)^ genotype mice.

For genotyping, the mouse tail was cut for approximately 0.2 cm in length and lysed for DNA extraction and served as the template for PCR by using the KAPA Mouse Genotyping Kit (Catalog No. KK7302, Merck, Darmstadt, Germany). The genotyping primers for transgenic APP were: 5′-AGGACTGACCACTCGACCAG-3′ (transgene forward), 5′-CGGGGGTCTAGTTCTGCAT-3′ (transgene reverse), 5′-CTAGGCCACAGAATTGAAAGATCT-3′ (internal positive control forward) and 5′-GTAGGTGGAAATTCTAGCATCATCC-3′ (internal positive control reverse). The genotyping primers for transgenic PS1 were: 5′-AATAGAGAACGGCAGGAGCA-3′ (transgene forward), 5′-GCCATGAGGGCACTAATCAT-3′ (transgene reverse), 5′-CTAGGCCACAGAATTGAAAGATCT-3′ (internal positive control forward) and 5′-GTAGGTGGAAATTCTAGCATCATCC-3′ (internal positive control reverse). The genotyping primers for Gal-3 were 5′-GACTGGAATTGCCCATGAAC-3′ (common forward), 5′-GAGGAGGGTCAAAGGGAAAG-3′ (wild-type reverse), and 5′-TCGCCTTCTTGACGAGTTCT-3′ (mutant reverse). The PCR product was separated by gel electrophoresis on a 1.5% agarose gel for genotype identification.

### Immunohistochemistry

The WT and APP/PS1 mice were anesthetized with pentobarbital (100 mg/kg, i.p.) and perfused with precold PBS followed by 4% paraformaldehyde. Brains were removed and postfixed in 20% sucrose/4% paraformaldehyde solution for 20–48 h. Frozen brains were cut into 30-μm sections on a cryostat and mounted on gelatin-coated slides. Brain sections were rinsed with 1× TBS for 10 min followed by antigen retrieved with 0.1 M citric acid/0.1 M sodium citrate buffer at 95 °C for 45 min. The sections were washed with 1× TBS for 10 min and permeabilization with 1× TBST (0.5% Triton X-100 in 1× TBS) for 10 min. Sections were preincubated in blocking solution (3% bovine serum albumin and 0.5% Triton X-100 in 1× TBS) for 1 h. For immunohistochemistry, brain sections were incubated with the following antibodies at 4 °C for overnight: goat anti-Gal-3 antibody (1:200, R&D), guinea pig anti-Iba1 antibody (1:200, Synaptic Systems, Goettingen, Germany), rabbit anti-GFAP antibody (1:200, Cell Signaling), and mouse anti-Aβ antibody 6E10 (1:500, BioLegend). For immunohistochemistry of Gal-3, brain sections were then washed with 1× TBS and incubated with donkey anti-goat secondary antibody conjugated with Alexa Fluor 488 (1:500, Jackson ImmunoResearch) for 1 h. For immunohistochemistry of Iba1, brain sections were incubated with Alexa Fluor® 594-conjugated donkey anti-guinea pig IgG antibody (1:200, Jackson ImmunoResearch) for 1 h. For immunohistochemistry of GFAP, brain sections were incubated with goat anti-rabbit secondary antibody conjugated with Cy3 (1:500, Jackson ImmunoResearch) for 1 h. For immunohistochemistry of Aβ, brain sections were incubated with goat anti-mouse secondary antibody conjugated with DyLight 549 (1:500, Jackson ImmunoResearch) for 1 h. For visualization of the nucleus, sections were mounted on slides with 15 μl of the DAPI Fluoromount-G® mounting medium (SouthernBiotech, Birmingham, AL) and washed by 1× TBS for 10 min for three times and stored at 4 °C. For visualization of the endogenously formed amyloid plaque in APP/PS1 mice, the PROTEOSTAT® Aggresome detection kit (Enzo Life Sciences, Farmingdale, NY) was used to detect amyloid plaque. Brain sections were subjected to 0.05% ProteoStat dye staining for 20 min and washed with ddH_2_O for 10 min for three times. Photomicrographs were taken using a Zeiss LSM700 Stage confocal microscope.

### Water maze learning

The water maze used was a plastic, circular pool, 1.2 m in diameter and 25 cm in height that was filled with water (25 ± 2 °C) to a depth of 16 cm. A circular platform of 8 cm in diameter was placed at a specific location away from the edge of the pool. The top of the platform was submerged 0.6 cm below the water surface. Water was made cloudy by adding milk powder. Distinctive, visual cues were set on the wall.

For spatial learning, animals were subjected to three trials a day with one given early in the morning, one given in the early afternoon and another one given in the late afternoon. The training procedure lasted for 5 days and a total of 15 trials were given. For these trials, animals were placed at different starting positions spaced equally around the perimeter of the pool in a random order. Animals were given 60 s to find the platform. If an animal could not find the platform, it was guided to the platform and was allowed to stay on the platform for 30 s. The time that each animal took to reach the platform was recorded as the escape latency. The total distance that animals traveled in the target region as well as their swim speed was also recorded. A probe trial of 60 s was given on day 6 to test their memory retention. Animals were placed in the pool with the platform been removed and the time they spent in each quadrant (target quadrant, left quadrant, opposite quadrant and right quadrant) was recorded.

## Patients

### Human frontal lobe lysate preparation

Human frontal lobe tissue lysate (GTX26550 for AD patients and GTX28727 for normal controls) were purchased from GeneTex, Inc. (Irvine, CA). Information about the tissue lysate is shown in Supplementary Table 2. The homogenized lysate (16 μg) was mixed with 5× protein loading buffer (GeneMark) before boiling at 95 °C for 10 min and was subjected to 13% tricine SDS-PAGE followed by western blotting. The procedures for western blot were the same as that described above for mice.

### Immunohistochemistry for human frontal lobe tissue

Human frontal lobe tissue slides (GTX24582 for AD patients and GTX24304 for normal controls) were purchased from GeneTex, Inc. Information about the tissue slides is shown in Supplementary Table 3. The slides were immersed with 100% xylene for 10 min twice for de-paraffinization followed by immersion in gradient ethanol (100%, 95%, 70 and 50%) once for 3 min for rehydratation. The sections were rinsed with 1× TBS for 10 min and antigen was retrieved with 0.1 M citric acid/0.1 M sodium citrate buffer at 95 °C for 45 min. The sections were washed with 1× TBS for 10 min for three times followed by immunohistochemical staining of Gal-3 using rabbit anti-Gal-3 antibody (1:200, Cell Signaling). For visualization of Gal-3, brain sections were then washed with 1× TBS and incubated with goat anti-rabbit secondary antibody conjugated with Alexa Fluor 488 (1:500, Jackson ImmunoResearch) for 1 h. The PROTEOSTAT® Aggresome detection kit (Enzo Life Sciences) was used to detect endogenous amyloid plaque as described above. Tissue sections were mounted with 15 μl of the DAPI Fluoromount-G® mounting medium (SouthernBiotech) for visualization of the nucleus as described above. Photomicrographs were taken using a Zeiss LSM700 Stage confocal microscope.

### Statistical analysis

All the data are presented as mean values ± SEM. Data were analyzed by Student’s *t* test or one-way analysis of variance (ANOVA) followed by Newman–Keuls multiple comparisons (represented by *q* value). Values of *P* < 0.05 are considered statistically significant (*P *<* 0.05, **P < 0.01, ^#^*P* < 0.001).

## Supplementary information

Supplementary Figure 1

Supplementary Figure 2

Supplementary Figure 3

Supplementary Figure 4

Supplementary Figure 5

Supplementary Figure 6

Supplementary Figure Legends

Supplementary Methods

Supplementary Table 1

Supplementary Table 2

Supplementary Table 3

## Data Availability

The original blots of all the western blot experiments are shown in Supplementary Fig. 6.
