# Supplementary figures and images for: Galectin-3 promotes Aβ oligomerization and Aβ toxicity in a mouse model of Alzheimer’s disease
Source: Cell Death Differ. 2019 May 24;27(1):192–209. doi: 10.1038/s41418-019-0348-z (PMC7206130; doi:10.1038/s41418-019-0348-z)

# Supplementary Figure 1

**a**

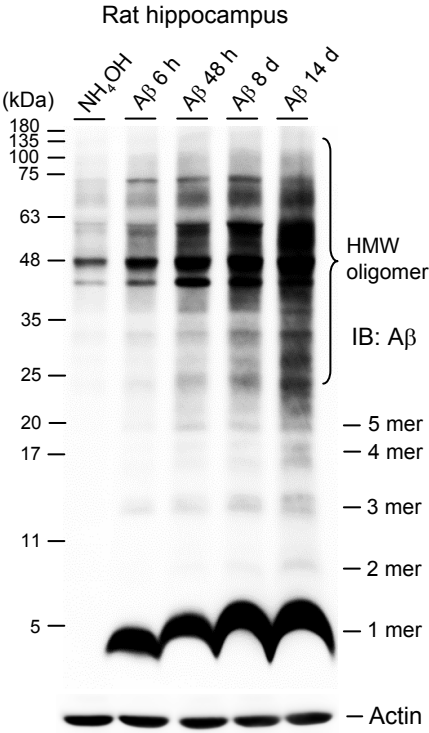

**b**

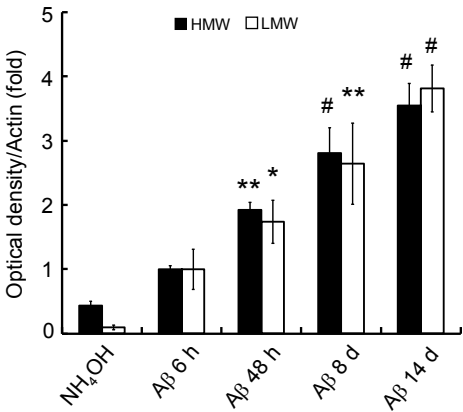

Supplement: Supplementary file 1 — Supplementary Figure 1 [file 41418_2019_348_MOESM1_ESM.pdf]

## Supplementary Figure 2

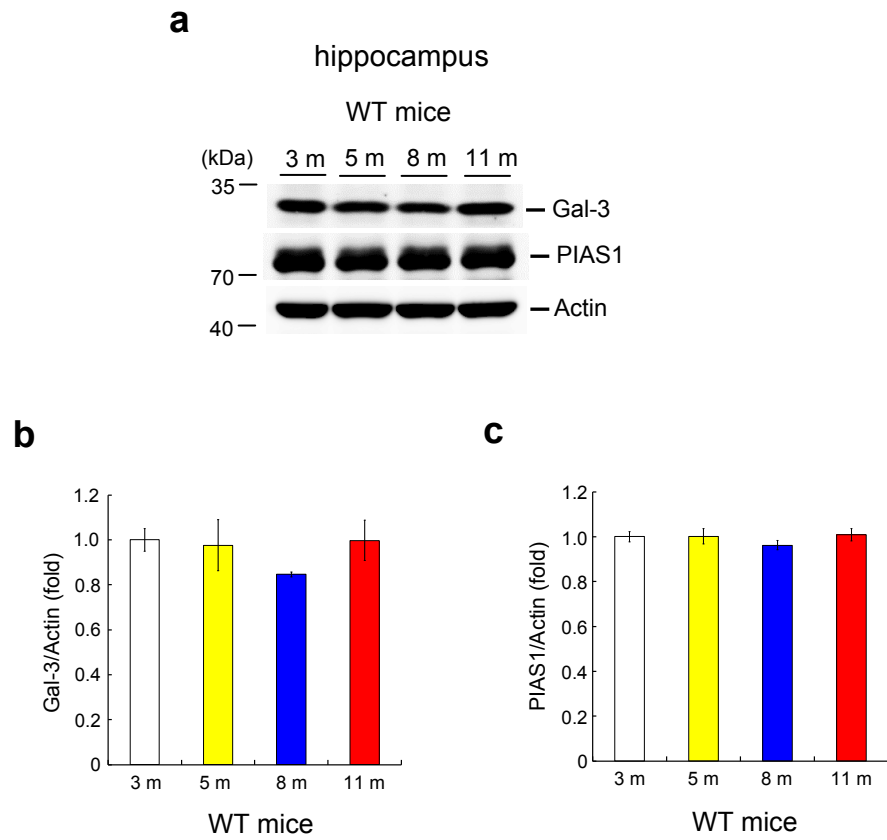

Supplement: Supplementary file 2 — Supplementary Figure 2 [file 41418_2019_348_MOESM2_ESM.pdf]

# Supplementary Figure 3

**a**

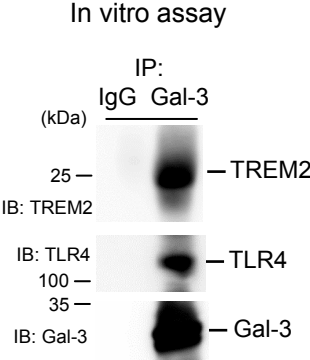

**b**

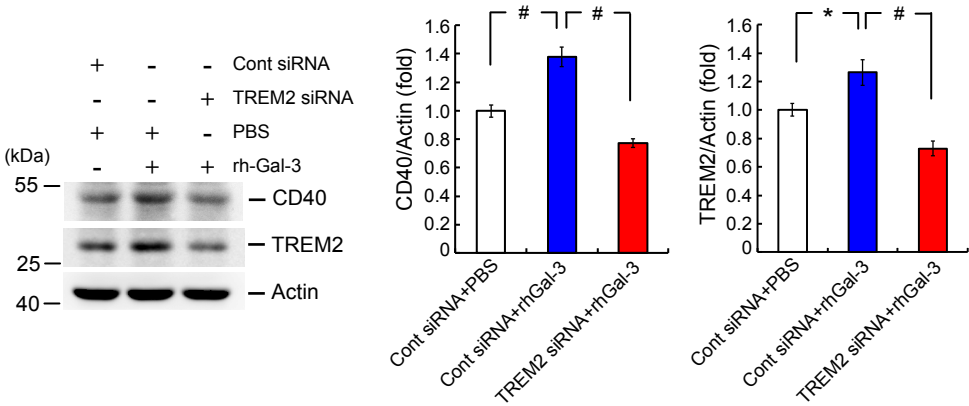

Supplement: Supplementary file 3 — Supplementary Figure 3 [file 41418_2019_348_MOESM3_ESM.pdf]

## Supplementary Figure 4

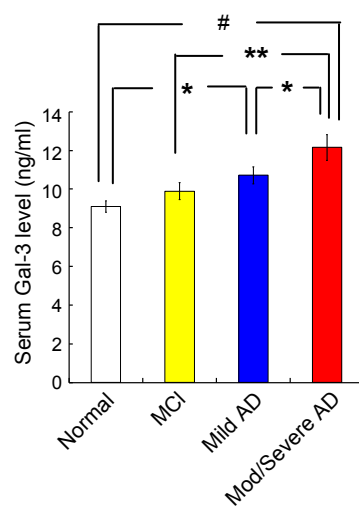

Supplement: Supplementary file 4 — Supplementary Figure 4 [file 41418_2019_348_MOESM4_ESM.pdf]

# Supplementary Figure 5

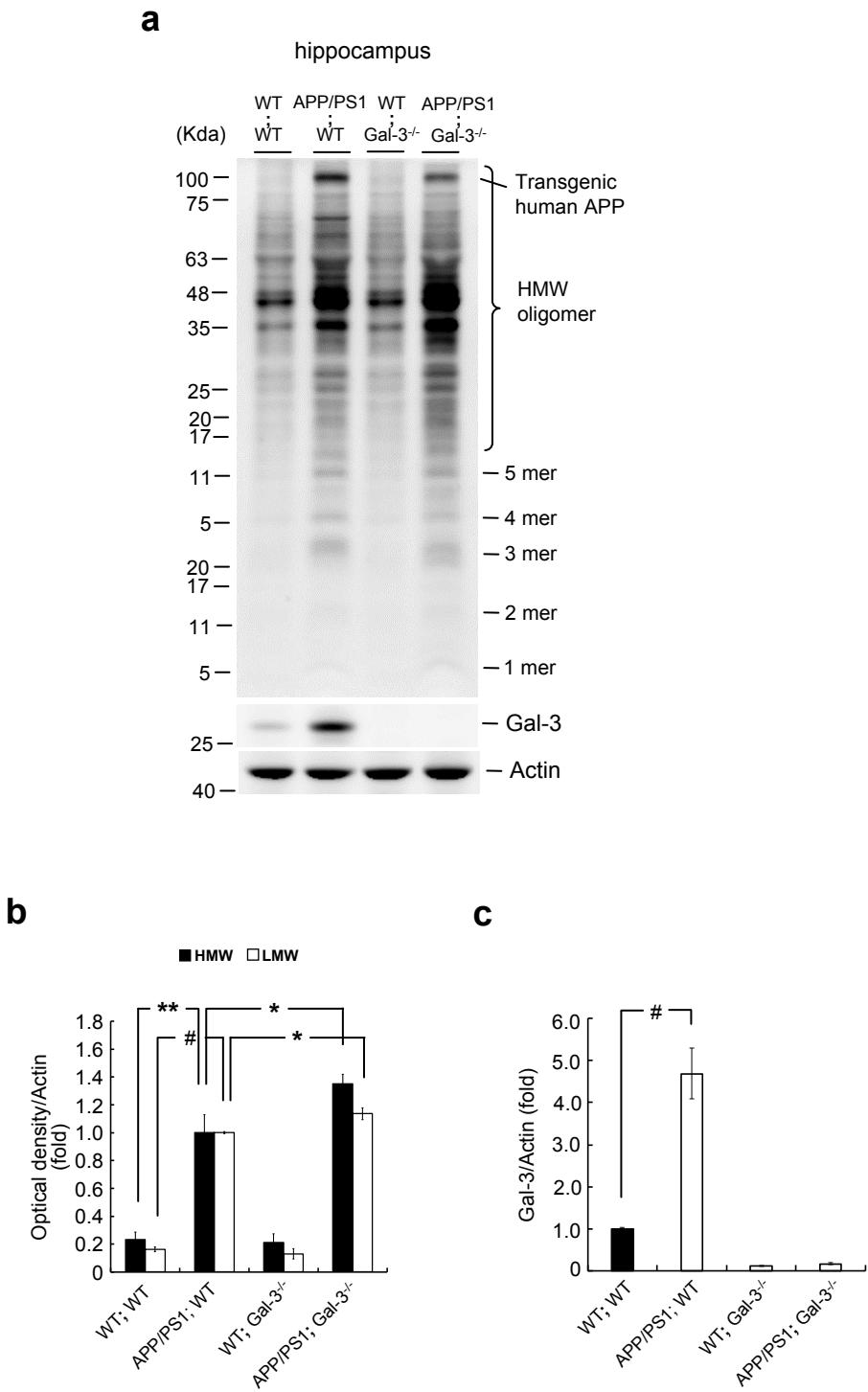

Supplement: Supplementary file 5 — Supplementary Figure 5 [file 41418_2019_348_MOESM5_ESM.pdf]
