# Supplementary material for: Galectin-3 promotes Aβ oligomerization and Aβ toxicity in a mouse model of Alzheimer’s disease
Source: Cell Death Differ. 2019 May 24;27(1):192–209. doi: 10.1038/s41418-019-0348-z (PMC7206130; doi:10.1038/s41418-019-0348-z)

Original gel blots for all the figures and supplementary figures

Figure 1

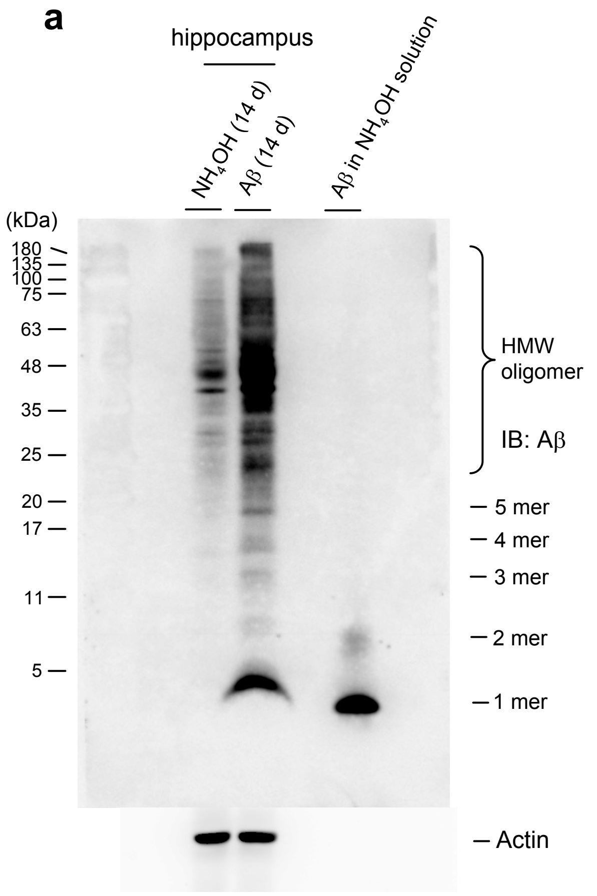

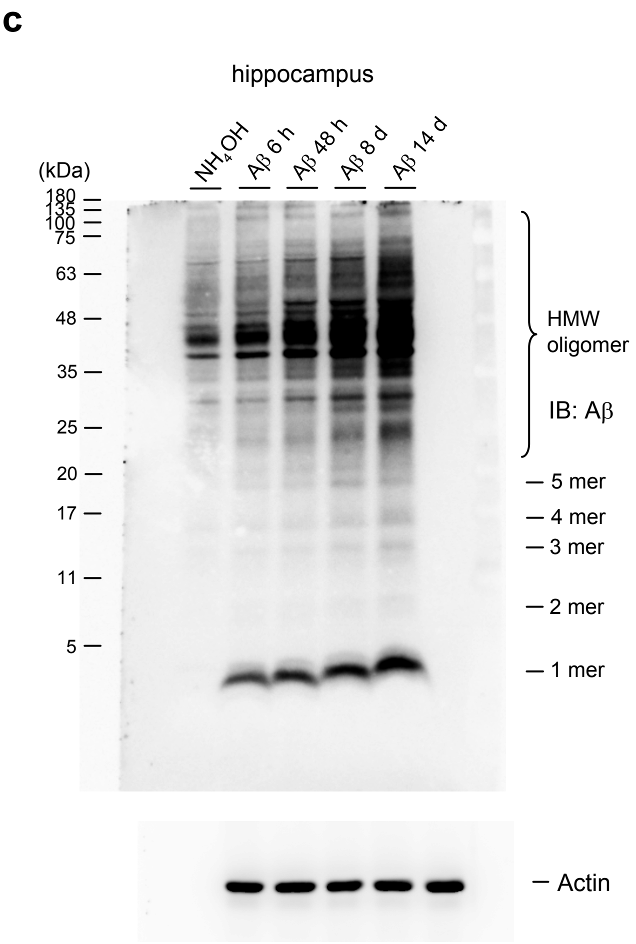

Figure 2

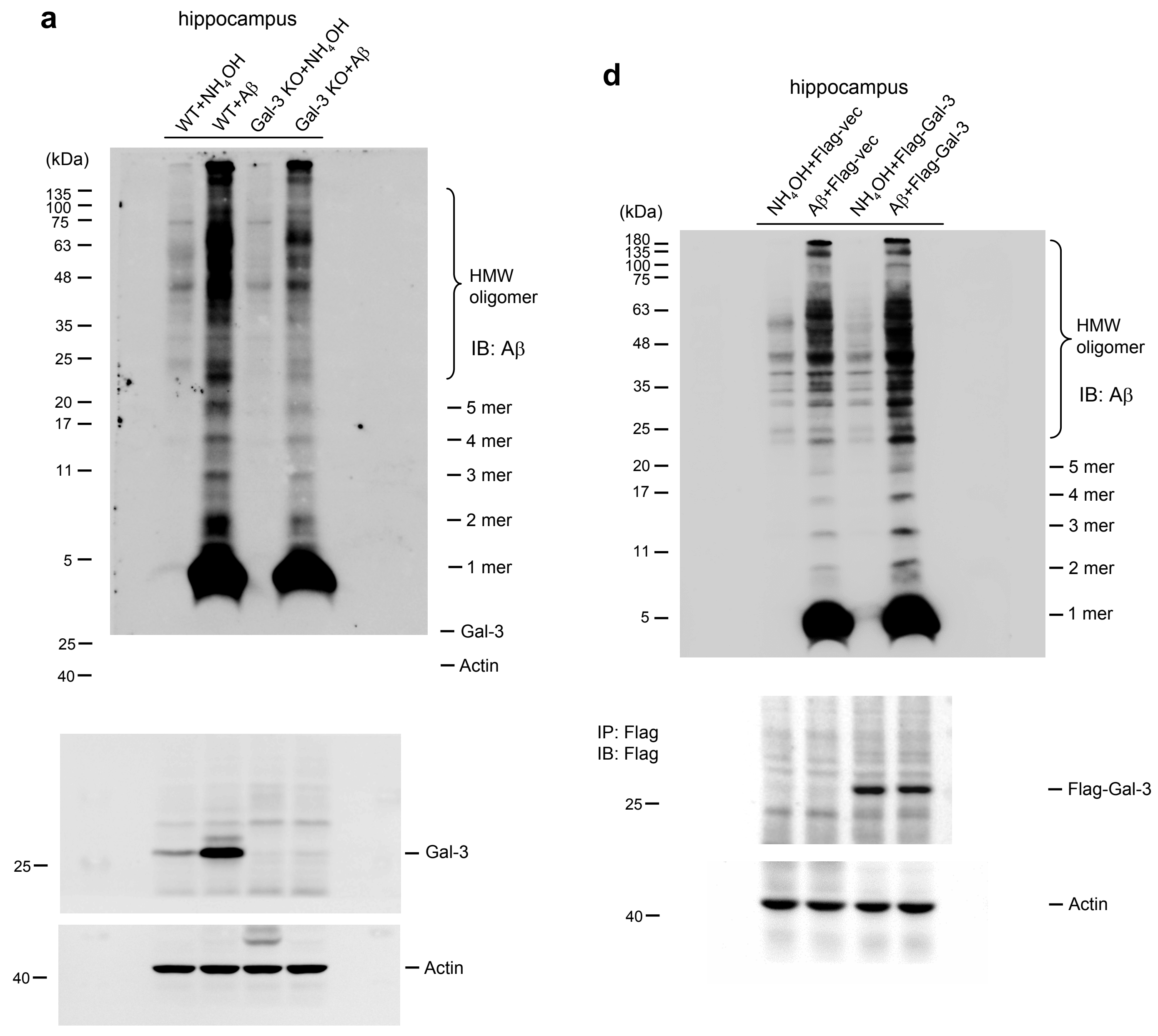
a
hippocampus
WT+NH4OH
WT+Aβ
Gal-3 KO+NH4OH
Gal-3 KO+Aβ
(kDa)
135
100
75
63
48
35
25
20
17
11
5
HMW oligomer
IB: Aβ
5 mer
4 mer
3 mer
2 mer
1 mer
Gal-3
Actin
25
40
d
hippocampus
NH4OH+Flag-vec
Aβ+Flag-vec
NH4OH+Flag-Gal-3
Aβ+Flag-Gal-3
(kDa)
180
135
100
75
63
48
35
25
20
17
11
5
HMW oligomer
IB: Aβ
5 mer
4 mer
3 mer
2 mer
1 mer
IP: Flag
IB: Flag
Flag-Gal-3
25
Actin
40

Figure 3

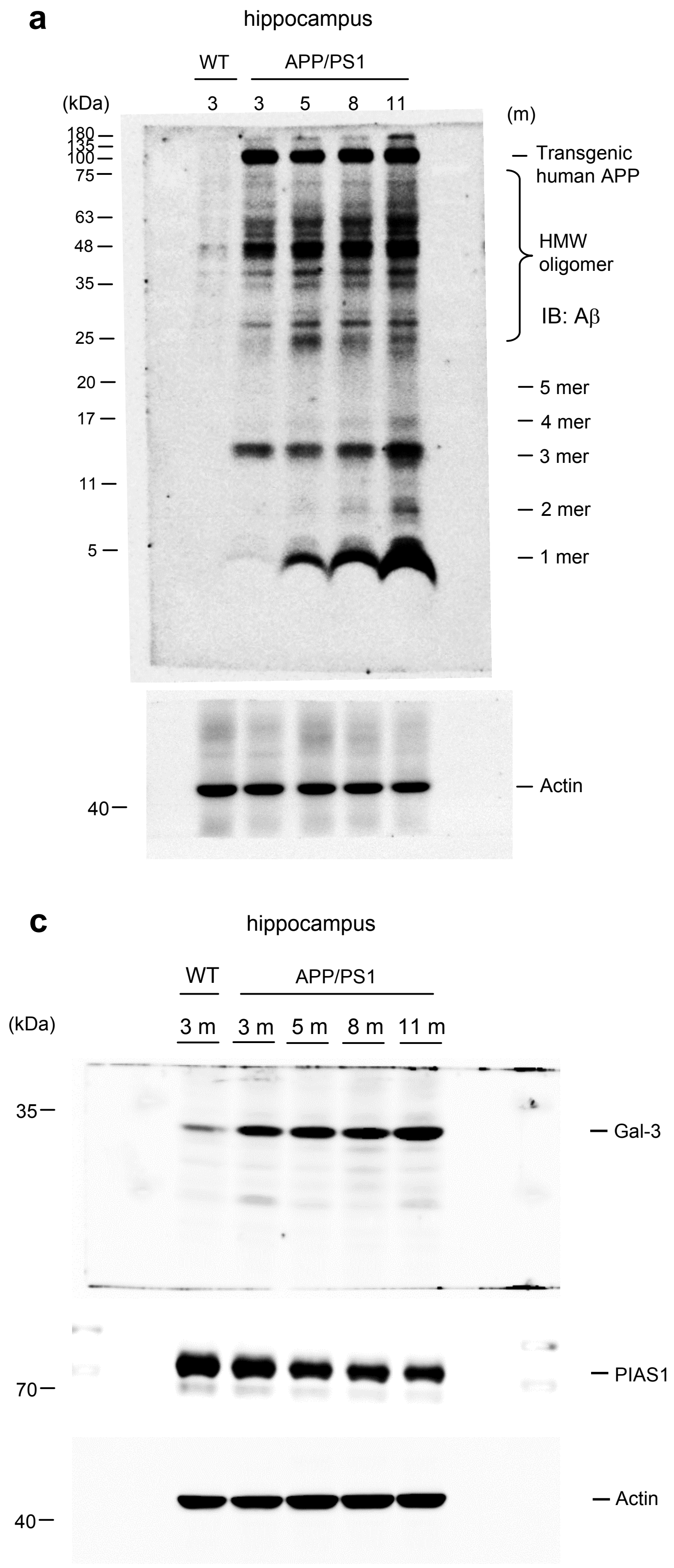

a
hippocampus
WT
APP/PS1
(kDa)
3 3 5 8 11
(m)
180
135
100
75
63
48
35
25
20
17
11
5
Transgenic human APP
HMW oligomer
IB: Aβ
5 mer
4 mer
3 mer
2 mer
1 mer
40
Actin
c
hippocampus
WT
APP/PS1
(kDa)
3 m 3 m 5 m 8 m 11 m
35
Gal-3
70
PIAS1
40
Actin

Figure 4

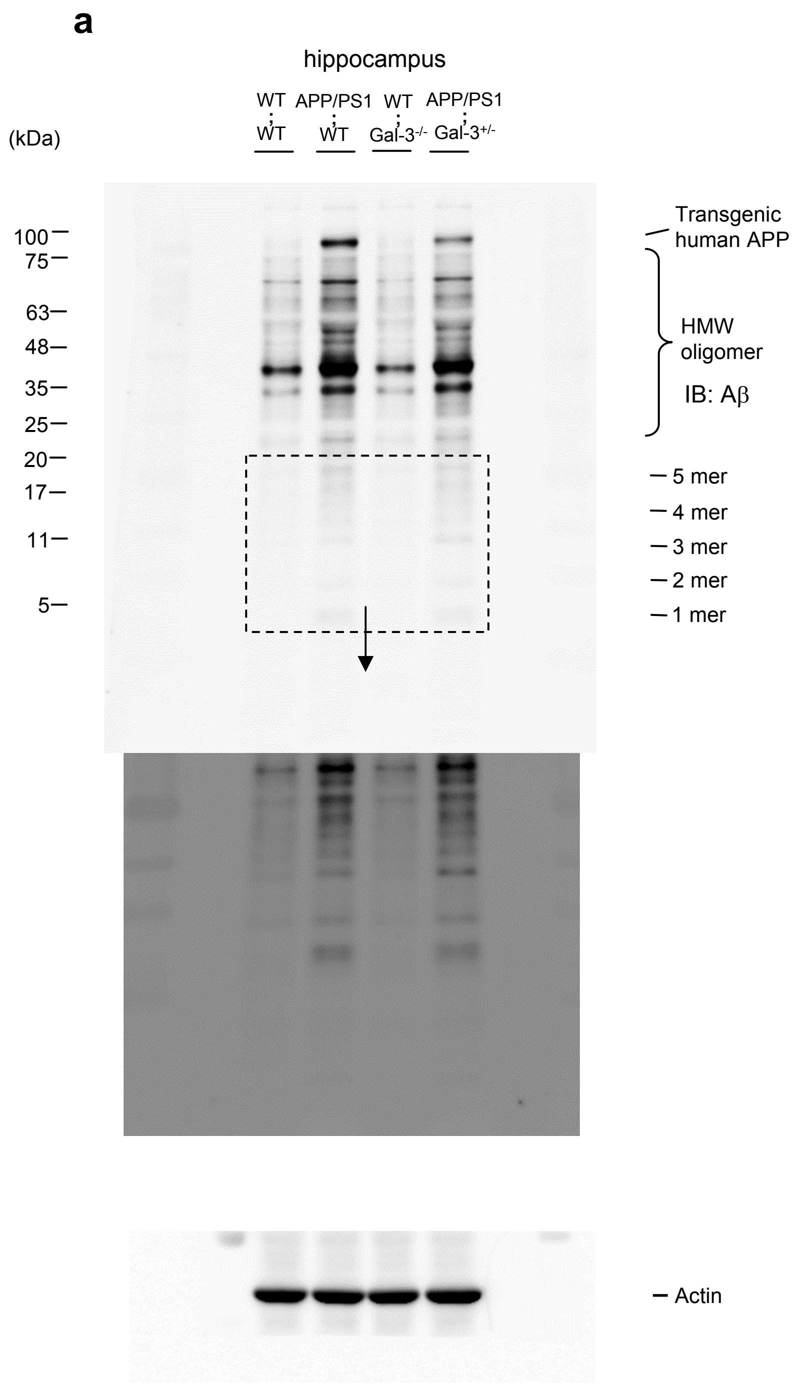

a
hippocampus
WT : WT
APP/PS1 : WT
WT : Gal-3-/-
APP/PS1 : Gal-3+/-
(kDa)
100
75
63
48
35
25
20
17
11
5
Transgenic human APP
HMW oligomer
IB: Aβ
5 mer
4 mer
3 mer
2 mer
1 mer
Actin

Figure 5

a

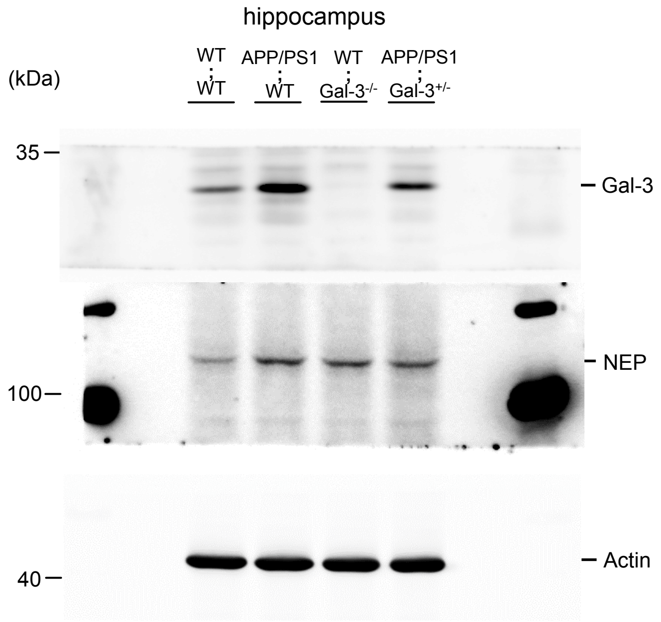

hippocampus
WT : WT
APP/PS1 : WT
WT : Gal-3-/-
APP/PS1 : Gal-3+/-
(kDa)
35
100
40
Gal-3
NEP
Actin

Figure 6

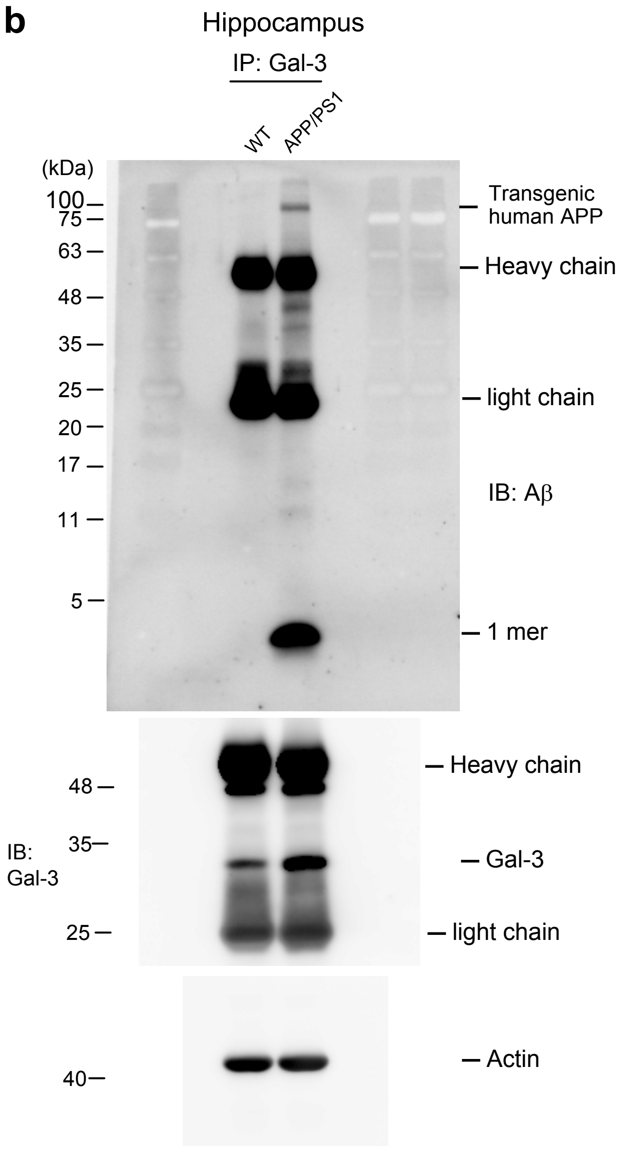
b
Hippocampus
IP: Gal-3
WT
APP/PS1
(kDa)
100
75
63
48
35
25
20
17
11
5
Transgenic human APP
Heavy chain
light chain
IB: Aβ
1 mer
48
35
25
IB: Gal-3
Heavy chain
Gal-3
light chain
40
Actin

Figure 7

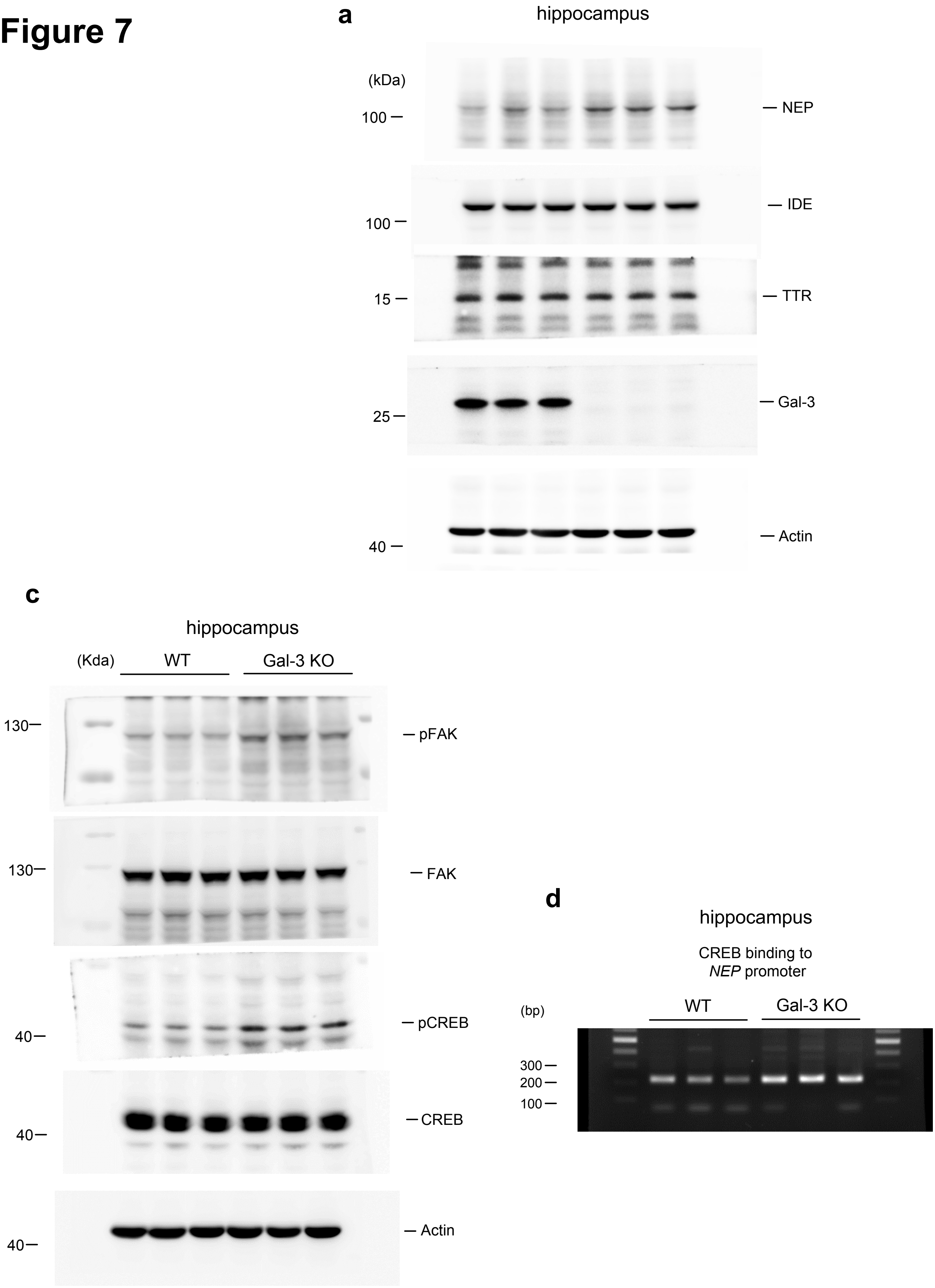
a
hippocampus
(kDa)
100
NEP
IDE
100
15
TTR
25
Gal-3
40
Actin
c
hippocampus
(Kda)
WT
Gal-3 KO
130
pFAK
130
FAK
40
pCREB
40
CREB
40
Actin
d
hippocampus
CREB binding to
NEP promoter
(bp)
WT
Gal-3 KO
300
200
100

Figure 8

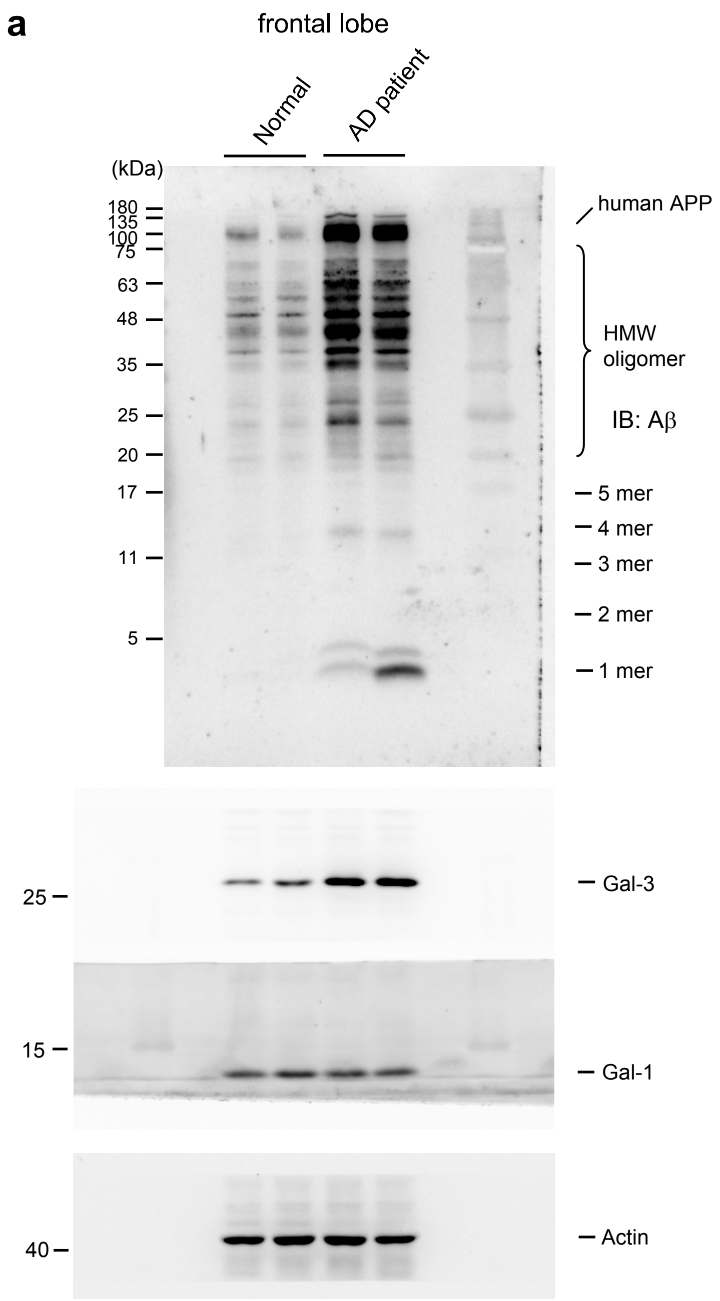
a
frontal lobe
Normal
AD patient
(kDa)
180
135
100
75
63
48
35
25
20
17
11
5
human APP
HMW
oligomer
IB: Aβ
5 mer
4 mer
3 mer
2 mer
1 mer
25
Gal-3
15
Gal-1
40
Actin

Supplementary Figure 1

a

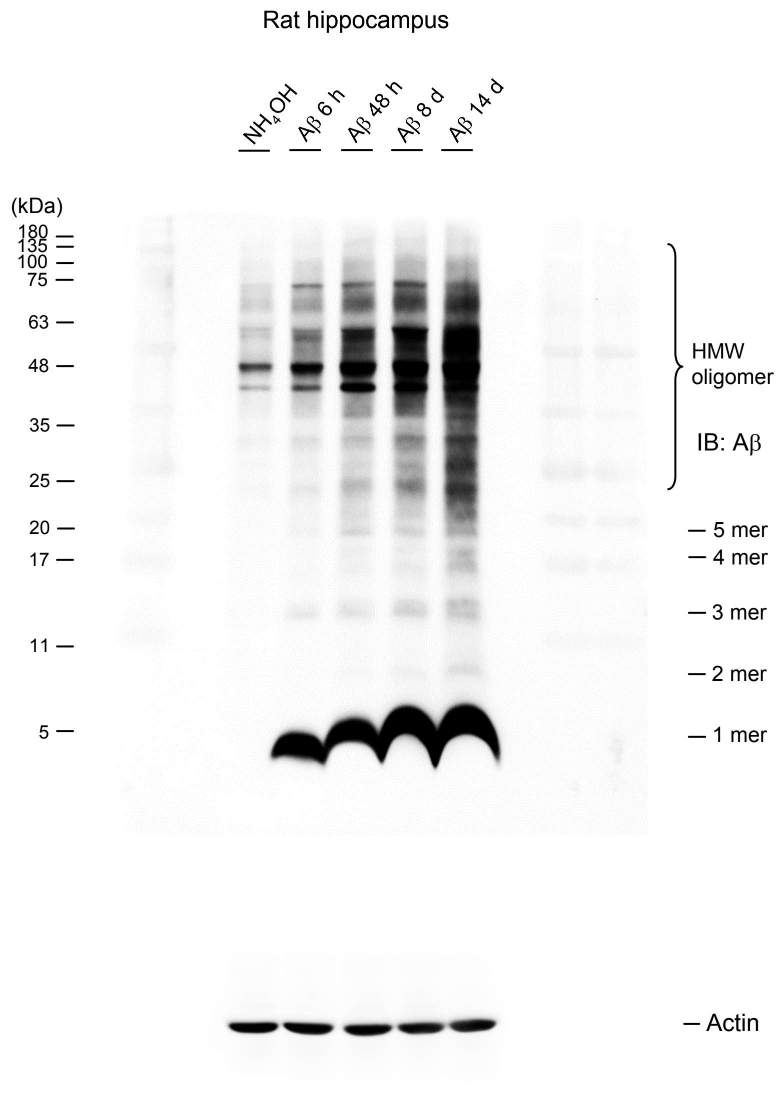

Supplementary Figure 2

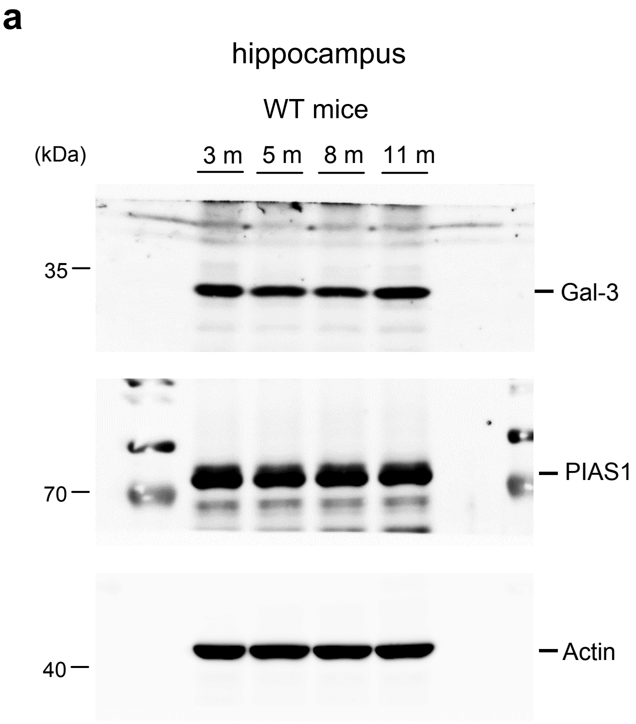

Supplementary Figure 3

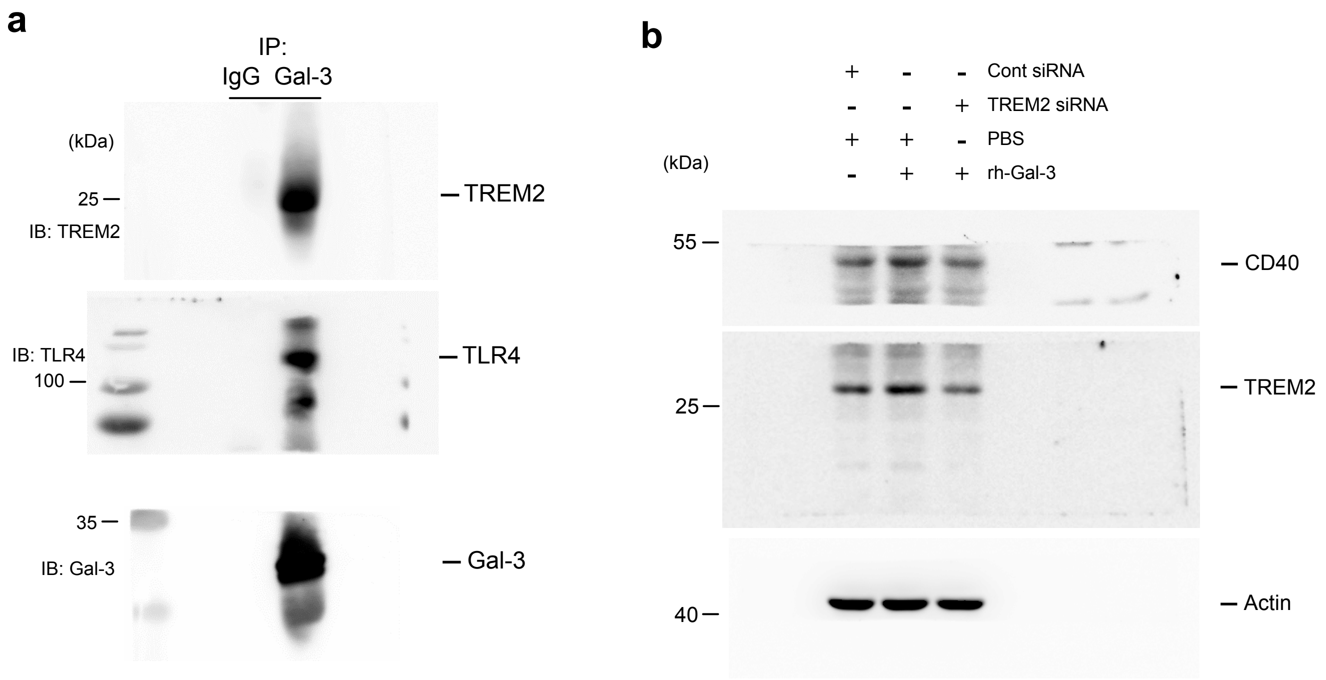

Raw data for Supplementary Figure 4 **Serum Gal-3 level (ng/ml)**

Control			MCI			
**no**	**Serum Gal-3**	**CV(%)**	**no**	**Serum Gal-3**	**CV(%)**	**no**
AD10501	**7.522**	1.020	ACM009	**8.276**	1.311	ACM956
AD10601	**9.814**	1.835	ACV114	**6.973**	0.702	ACT198
AD10405	**6.256**	2.627	ACT099	**11.076**	0.440	ACM955
AD10502	**7.387**	1.157	ACM975	**8.176**	0.675	ACU779
AD10402	**6.954**	0.800	ACM970	**6.947**	0.961	ACM001
AD10401	**8.889**	1.165	ACM915	**13.515**	0.235	ACT055
ACV676	**9.146**	0.052	ACN010	**4.211**	2.779	ACM926
dem468	**10.057**	0.076	ACN032	**12.476**	0.768	ACU548
ACV058	**6.559**	0.130	ACT363	**12.617**	0.063	ACN002
dem430	**8.148**	0.190	ACN212	**9.581**	0.165	ACU269
dem551	**7.558**	0.222	ACU669	**10.450**	0.257	ACT222
dem506	**9.327**	0.339	ACV329	**5.182**	0.301	ACU672
dem345	**4.678**	0.346	ACV904	**7.758**	0.345	ACT026
dem493	**14.119**	0.414	ABR497	**7.868**	0.351	ACN729
dem434	**10.890**	0.416	ACM084	**7.943**	0.419	ACN715
dem534	**7.813**	0.454	ACN082	**12.627**	0.439	ACT115
dem517	**7.705**	0.596	ABR432	**9.898**	0.481	ACN811
dem488	**5.686**	0.613	ACN249	**9.141**	0.562	ACT040
dem495	**9.234**	0.642	ACN784	**9.331**	0.575	ABR436
dem496	**9.261**	0.662	ACN855	**10.177**	0.597	ACV900
dem494	**10.623**	0.833	ACN307	**12.297**	0.661	ACT482
dem314	**11.550**	0.838	ACT628	**8.469**	0.858	ACN288
dem490	**9.551**	0.935	dem316-F	**17.334**	0.911	ACV180
dem519	**7.309**	0.944	ACU264	**13.551**	0.934	ACN460
dem450	**13.066**	0.971	ABR380	**9.605**	0.949	ACN756
dem543	**10.103**	0.983	ACN813	**8.502**	0.970	ACN737
dem350	**6.041**	1.051	ACV665	**9.901**	1.062	ACU161
dem466	**9.311**	1.070	ACN086	**8.874**	1.068	ACV158
ACT010	**13.605**	1.217	ABR494	**8.395**	1.080	ACU418
ACV514	**6.435**	1.228	ACN243	**4.718**	1.163	ACM024
dem319	**9.708**	1.302	ACN191	**10.514**	1.226	ACN493
dem443	**8.681**	1.332	ACN105	**12.775**	1.238	ACU751
dem508	**10.366**	1.345	ACV291	**14.351**	1.281	ACU049
dem557	**10.756**	1.360	ACV567	**12.674**	1.327	ACT340
dem504	**8.811**	1.677	dem319-F	**11.769**	1.487	ACN786
dem478	**9.921**	1.794	ACM340	**8.661**	1.513	ACT745
ACU592	**8.518**	1.840	ACN150	**15.409**	1.535	ACV214
dem463	**11.644**	1.846	lem350-F1	**6.421**	1.601	ACU053
dem315	**10.496**	1.867	ACN694	**9.048**	1.615	ACV049

ACV294	**8.537**	2.264
dem272	**11.085**	2.853
dem526	**5.930**	3.152
dem424	**11.424**	3.427
ACM072	**8.057**	3.917
dem341	**8.621**	3.933
dem542	**11.904**	4.215
dem505	**7.558**	5.427
ACU153	**10.030**	6.576

ACN438	**16.839**	1.779
ACN814	**8.491**	2.181
ACV471	**11.388**	2.478
ACN157	**9.301**	2.963
ACN237	**6.186**	3.074
ACN252	**8.005**	3.216
ACN066	**7.012**	3.381

ACU545
ACN797
ACN449
ACU971
ACT513
ACU558
ACT410
ACN189
ACT717
ACU110
ACV874
ACN369
ACU111

mild AD		moderate and severe AD		
Serum Gal-3	CV(%)	no	Serum Gal-3	CV(%)
**7.246**	2.011	ACN046	**12.191**	0.575
**15.399**	0.290	ACV148	**20.181**	0.081
**14.911**	0.479	ACN058	**7.443**	1.847
**11.403**	0.023	ACT212	**11.840**	0.359
**6.503**	2.709	ACU172	**21.144**	0.902
**9.348**	0.461	ACU137	**12.237**	0.442
**10.028**	0.294	ACN014	**12.417**	0.308
**8.329**	0.472	ACM954	**10.280**	1.061
**9.161**	0.415	ACM930	**10.419**	0.287
**7.586**	0.052	ACN282	**23.447**	0.020
**11.691**	0.064	ACT234	**8.932**	0.172
**14.366**	0.088	ACN335	**13.764**	0.181
**11.106**	0.144	ABR376	**13.629**	0.334
**12.587**	0.195	ACM056	**11.254**	0.464
**9.623**	0.253	ABR355	**12.427**	0.590
**9.192**	0.266	ACN125	**16.086**	0.597
**12.815**	0.306	ACN280	**12.394**	0.598
**5.228**	0.372	ACT409	**18.643**	0.672
**13.689**	0.455	ABR495	**9.652**	0.862
**16.129**	0.459	ACN775	**11.885**	0.882
**11.406**	0.489	ABR501	**10.299**	0.928
**7.960**	0.529	ACN301	**7.389**	0.952
**15.634**	0.574	ACN841	**6.590**	0.957
**10.726**	0.708	ACT829	**9.048**	0.994
**14.567**	0.763	ACN348	**9.428**	1.036
**10.830**	0.838	ACN473	**6.154**	1.066
**7.843**	0.976	ACN733	**9.726**	1.334
**19.171**	1.049	ACN245	**12.650**	1.418
**10.296**	1.180	ACN711	**10.545**	1.475
**16.953**	1.349	ACU014	**9.725**	1.536
**8.772**	1.351	ABR423	**6.581**	1.557
**7.578**	1.364	ABR473	**8.453**	1.673
**17.295**	1.409	ABR440	**19.193**	1.709
**9.551**	1.410	ACV382	**16.809**	1.882
**6.867**	1.489	ACT594	**10.902**	1.908
**9.358**	1.676	ACN330	**7.104**	2.056
**7.553**	1.984	ACU117	**11.904**	2.312
**10.240**	2.047	ACV635	**18.142**	2.599
**9.623**	2.194	ACN349	**12.344**	2.760

**10.496**	2.317
**12.504**	2.609
**9.670**	2.841
**9.563**	3.123
**6.693**	3.294
**8.357**	3.657
**6.579**	3.972
**9.716**	4.007
**15.020**	4.032
**9.321**	4.271
**14.133**	4.346
**7.714**	6.456
**8.571**	8.996

ACN334	**25.145**	2.934
ACJ014	**11.206**	3.095
ACN318	**8.254**	3.107
ACN661	**8.016**	3.419
ACN697	**11.323**	8.319
ACU359	**9.438**	8.877

Supplementary Figure 5

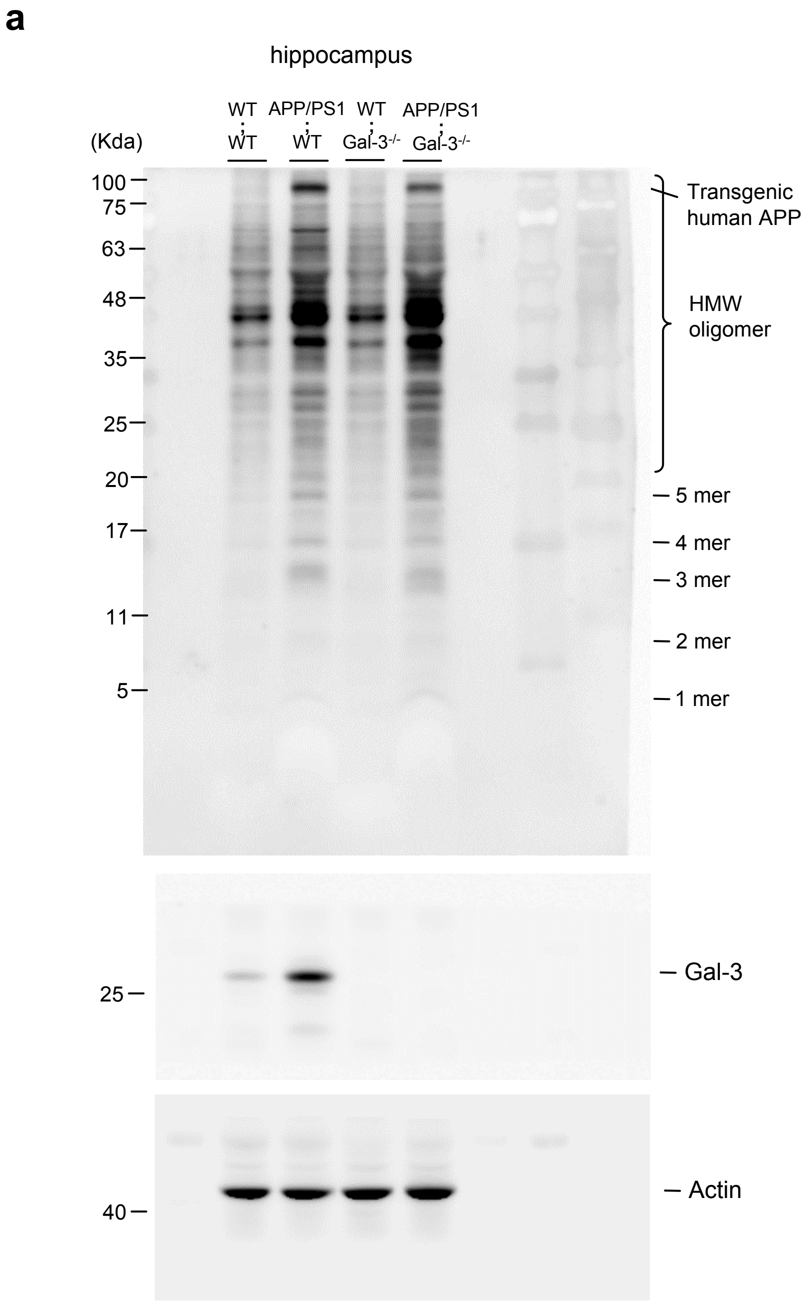

Supplement: Supplementary file 6 — Supplementary Figure 6 [file 41418_2019_348_MOESM6_ESM.pdf]
