# Supplementary material for: Galectin-3 promotes Aβ oligomerization and Aβ toxicity in a mouse model of Alzheimer’s disease
Source: Cell Death Differ. 2019 May 24;27(1):192–209. doi: 10.1038/s41418-019-0348-z (PMC7206130; doi:10.1038/s41418-019-0348-z)
Supplement: Supplementary file 7 — Supplementary Figure Legends [file 41418_2019_348_MOESM7_ESM.pdf]

Supplementary Figure Legends

Figure S1. Intra-hippocampal Aβ injection causes Aβ oligomerization in rats.

(a) 1% NH_4OH or Aβ (14 μg) was injected to the CA1 area of rats (3-month old) and they were sacrificed at different time periods (6 h, 48 h, 8 days and 14 days) after Aβ injection. Their dorsal hippocampal tissue was dissected out and subjected to western blot analysis of Aβ oligomerization. **(b)** The quantified result of HMW and LMW Aβ oligomerization is shown (n=3 each group) ($F(4,10) = 27.19$, $P < 0.001$ for HMW and ($F(4,10) = 14.02$, $P < 0.001$ for LMW). Data are expressed as mean ± SEM. Statistical significance of various sets of comparisons is shown in the figure. * $P < 0.05$, ** $P < 0.01$, # $P < 0.001$ compared with the control group.

Figure S2. Galectin-3 and PIAS1 expression is not altered in different ages of WT mice.

(a) The hippocampal tissue from different ages (3 months, 5 months, 8 months and 11 months) of WT mice was dissected out and subjected to western blot analysis of Gal-3 and PIAS1 expression. **(b)** The quantified result of Gal-3 expression is shown (n=4 each group) ($F(3,12) = 0.9$, $P > 0.05$). **(c)** The quantified result of PIAS1 expression is shown (n=4 each group) ($F(3,12) = 0.62$, $P > 0.05$). m: month. Data are expressed as mean ± SEM.

Figure S3. TREM2 mediates the effect of Gal-3 on microglia activation.

(a) Recombinant TREM2 and TLR4 proteins were added to the recombinant Gal-3 protein, co-IP was carried out with anti-Gal-3 (or anti-IgG) antibody and immunoblotting was performed with anti-TREM2, anti-TLR4 and anti-Gal-3 antibodies. Experiments were performed in duplicate. **(b)** BV-2 cells were treated with recombinant human Gal-3 protein (100 nM dissolved in PBS) and transfected with TREM2 siRNA (100 nM) or control siRNA. In some groups, PBS was added to equalize the reaction volume. The expression levels of CD40 and TREM2 were determined by Western blot analysis and the results were quantified [n=4 per group; for CD40, $F(2,9) = 37.39$, $P < 0.001$, $q = 7.6$, $P < 0.001$ for the control siRNA+rhGal-3 group versus the control siRNA+PBS group; $q = 12.1$, $P < 0.001$ for the control siRNA+rhGal-3 group versus the TREM2 siRNA+rhGal-3 group; for TREM2, $F(2,9) = 16.54$, $P < 0.001$, $q = 3.99$, $P < 0.05$ for the control siRNA+rhGal-3 group versus the control siRNA+PBS group and $q = 8.13$, $P < 0.001$ for the control siRNA+rhGal-3 group versus the TREM2 siRNA+rhGal-3 group]. Data are expressed as mean ± SEM. * $P < 0.05$, # $P < 0.001$.

Figure S4. Serum Gal-3 level is increased as the severity of memory loss is

increased in AD patients.

Serum Gal-3 level was determined by ELISA in normal subjects (n=48) and in patients diagnosed as mild cognitive impairment (MCI) (n=46), mild AD (n=52) and moderate/severe AD (n=45) ($F(3,187) = 7.25$, $P < 0.001$). Statistical significance of various sets of comparisons is shown in the figure. Data are expressed as mean ± SEM. * $P < 0.05$, ** $P < 0.01$, # $P < 0.001$.

Figure S5. Endogenous Aβ oligomerization is increased in APP/PS1;WT and APP/PS1;Gal-3 KO mice.

(a) Endogenous Aβ oligomerization was examined by western blot in four genotypes of mice at 3-month old: WT;WT, APP/PS1;WT, WT;Gal-$3^{-/-}$ and APP/PS1;Gal-$3^{+/-}$ mice. Gal-3 expression was also determined by western blot. **(b)** The quantified result of HMW and LMW Aβ oligomerization is shown (n=2 each group) ($F(3,4) = 45.18$, $P < 0.01$ for HMW and ($F(3,4) = 322.38$, $P < 0.001$ for LMW). Statistical significance of various sets of comparisons is shown in the figure. **(c)** The quantified result of Gal-3 expression in the same groups of animals is shown ($F(3,4) = 51.01$, $P = 0.001$; $q = 12.16$, $P < 0.001$ comparing the APP/PS1;WT group with WT;WT group). Data are expressed as mean ± SEM. * $P < 0.05$, ** $P < 0.01$, # $P < 0.001$.
