# Supplementary material for: Galectin-3 promotes Aβ oligomerization and Aβ toxicity in a mouse model of Alzheimer’s disease
Source: Cell Death Differ. 2019 May 24;27(1):192–209. doi: 10.1038/s41418-019-0348-z (PMC7206130; doi:10.1038/s41418-019-0348-z)
Supplement: Supplementary file 8 — Supplementary Methods [file 41418_2019_348_MOESM8_ESM.pdf]

Supplementary Methods

Cell culture, small interfering RNA (siRNA) transfection and recombinant human Gal-3 treatment. BV-2 cells were maintained in Dulbecco's modified Eagle's medium containing 5 % fetal bovine serum and were incubated at 37 °C in a humidified atmosphere with 5 % CO_2. TREM2 siRNA transfection (100 nM/well) was made by using the Lipofectamine 2000 reagent (Invitrogen) in 12-well culture plates according to the manufacturer's instructions. The sequence for mouse TREM2 siRNA sense strand is: 5′-GAGGGUGUCAUGUACUUAUdTdT-3′ and that for antisense strand is: 5′-AUAAGUACAUGACACCCUCtt-3′. TREM2 siRNA was synthesized from Biotools (New Taipei City, Taiwan). The Silencer Negative Control number 1 siRNA (Ambion®, Thermo Fisher Scientific, Waltham, MA) was used as the control. Culture medium was replaced with the fresh growth medium containing 100 nM recombinant human Gal-3 (Catalog No. 8259-GA-050, R&D systems, Minneapolis, MN) 24 h after siRNA transfection. Culture medium added with equal volume of PBS was used as a control. Twenty-four hour after Gal-3 treatment, BV-2 cells were lysed with RIPA buffer and were subjected to western blot assays using 12% Tris-Glycine SDS-PAGE.

Participants

Normal healthy elderly, mild cognitive impairment (MCI) patients and Alzheimer's disease (AD) patients were recruited from the Taipei Veterans General Hospital and Taichung Veterans General Hospital in Taiwan. An AD diagnosis was made during a multidisciplinary consensus meeting according to the clinical criteria for probable AD as described by the National Institute on Aging-Alzheimer's Association.[49] A diagnosis of MCI was made according to the revised consensus criteria from 2004.[50] The cut-off value for diagnosis of MCI was set at 1.5 standard deviations below the age-adjusted norm for the logical memory test of the Wechsler Memory Scale III. All patients received a standardized evaluation that included a clinical interview, neuropsychological assessment, laboratory tests and brain magnetic resonance imaging. Informed consent was obtained from all patients and their caregivers before study participation.

Clinical evaluation and procedures

Cognitive function was assessed by a standard neuropsychological battery. The Mini-mental status examination (MMSE) was used to assess global cognition. The Clinical dementia rating (CDR) was administered to determine the severity of dementia.

DNA analysis

Genomic DNA was isolated from whole blood using a GentraPuregene kit according to the manufacturer's protocol (Qiagen, Hilden, Germany). The presence of the ε2, ε3, and ε4 alleles of the APOE (Apolipoprotein) gene were determined by genotyping of SNPs rs429358 and rs7412. The APOE ε4 carrier was defined as having at least one ε4 allele (including ε2/ε4, ε3/ε4, and ε4/ε4). Genotyping of rs429358 and rs7412 was performed using the TaqMan genotyping assay (Applied Biosystems, Foster City, CA). Polymerase chain reactions were performed in 96-well microplates with an ABI 7500 real-time PCR machine (Applied Biosystems). Allele discrimination was achieved by detecting fluorescence using System SDS software version 1.2.3 (Applied Biosystems).

Serum Gal-3 level measurement

After centrifuging the venous blood clotted in a serum separator tube, serum was separated and stored in polypropylene tubes at -80°C until biochemical analysis. Serum Gal-3 levels were measured in duplicate using enzyme linked immunosorbent assay kits (R&D Systems, Quantikine® ELISA, Minneapolis, Minnesota) following the manufacturer's instructions and standard procedures. The optical density (OD) was determined by the absorbance was measured at 450 nm and 540 nm (subtract

readings at 540 nm from the readings at 450 nm). We averaged the duplicate readings for each standard and sample and subtracted the average zero standard OD. The standard curve was created using the average standard values and each serum Gal-3 level was calculated form the average OD value according to the standard curve.
