# Supplementary material for: Galectin-3 promotes Aβ oligomerization and Aβ toxicity in a mouse model of Alzheimer’s disease
Source: Cell Death Differ. 2019 May 24;27(1):192–209. doi: 10.1038/s41418-019-0348-z (PMC7206130; doi:10.1038/s41418-019-0348-z)
Supplement: Supplementary file 9 — Supplementary Table 1 [file 41418_2019_348_MOESM9_ESM.pdf]

Supplementary Table 1

Swim speed of WT;WT, APP/PS1;WT, WT;Gal-$3^{-/-}$ and APP/PS1;Gal-$3^{+/-}$ mice from the retention test of water maze learning

Swim speed (cm/sec)			
WT;WT	APP/PS1;WT	WT;Gal-$3^{-/-}$	APP/PS1;Gal-$3^{+/-}$
17.65 ± 0.71	19.26 ± 0.81	18.14 ± 1.26	19.76 ± 0.83

$F(3,24) = 1.11$, $P > 0.05$. N = 7 each group.

Data are expressed as mean ± SEM.
