# Supplementary material for: Galectin-3 promotes Aβ oligomerization and Aβ toxicity in a mouse model of Alzheimer’s disease
Source: Cell Death Differ. 2019 May 24;27(1):192–209. doi: 10.1038/s41418-019-0348-z (PMC7206130; doi:10.1038/s41418-019-0348-z)
Supplement: Supplementary file 10 — Supplementary Table 2 [file 41418_2019_348_MOESM10_ESM.pdf]

Supplementary Table 2

Description of frontal lobe tissue lysate from human samples

Catalog #	Description	Donor #	Size	Concen. (mg/ml)	Age	Gender	Cause of Death
GTX28727	Human Brain: Frontal Lobe (Normal) tissue lysate	1	1 mg	5	78	F	Congestive heart failure
		2	1 mg	5	66	M	Arrhythmia, Possible Myocardial infarct
		3	1 mg	5	70	F	Breast cancer
		4	1 mg	5	77	M	lung cancer
		5	1mg	5	87	F	Asthma Chronic Obstructive Pulmonary Disease
GTX26550	Human Brain: Frontal Lobe (Alzheimer's disease) tissue lysate	1	1 mg	5	83	M	Alzheimer/renal failure
		2	1 mg	5	87	M	Alzheimer, prostate cancer
		3	1 mg	5	80	M	Alzheimer
		4	1 mg	5	80	M	N/A
		5	1 mg	5	85	F	Alzheimer
