# Supplementary material for: Galectin-3 promotes Aβ oligomerization and Aβ toxicity in a mouse model of Alzheimer’s disease
Source: Cell Death Differ. 2019 May 24;27(1):192–209. doi: 10.1038/s41418-019-0348-z (PMC7206130; doi:10.1038/s41418-019-0348-z)
Supplement: Supplementary file 11 — Supplementary Table 3 [file 41418_2019_348_MOESM11_ESM.pdf]

Supplementary Table 3

Description of frontal lobe tissue slides from human samples

Catalog #	Description	Donor #	Size	Age	Gender	Cause of Death
GTX24304	Human Brain: Frontal Lobe (Normal) tissue slides	1	5 slides	36	M	Accident
		2	5 slides	54	M	pancreas tumor
		3	5 slides	54	F	Hodgkin's Lymphoma
GTX24582	Human Brain: Frontal Lobe (Alzheimer's disease) tissue slides	1	5 slides	72	M	Alzheimer
		2	5 slides	73	M	Alzheimer
		3	5 slides	85	F	Alzheimer
